# Dynamical Behavior of β-Lactamases and Penicillin- Binding Proteins in Different Functional States and Its Potential Role in Evolution

**DOI:** 10.3390/e21111130

**Published:** 2019-11-19

**Authors:** Feng Wang, Hongyu Zhou, Xinlei Wang, Peng Tao

**Affiliations:** 1 Department of Chemistry, Center for Drug Discovery, Design, and Delivery (CD4), Center for Scientific Computation, Southern Methodist University, Dallas, TX 75275, USA; fengw@smu.edu (F.W.); hongyuz@smu.edu (H.Z.); 2 Department of Statistical Science, Southern Methodist University, Dallas, TX 75275, USA; swang@smu.edu

**Keywords:** TEM-1, TOHO-1, PBP-A, DD-transpeptidase, conformational changes, catalytic mechanism, evolution

## Abstract

β-Lactamases are enzymes produced by bacteria to hydrolyze β-lactam-based antibiotics, and pose serious threat to public health through related antibiotic resistance. Class A β-lactamases are structurally and functionally related to penicillin-binding proteins (PBPs). Despite the extensive studies of the structures, catalytic mechanisms and dynamics of both β-lactamases and PBPs, the potentially different dynamical behaviors of these proteins in different functional states still remain elusive in general. In this study, four evolutionarily related proteins, including TEM-1 and TOHO-1 as class A β-lactamases, PBP-A and DD-transpeptidase as two PBPs, are subjected to molecular dynamics simulations and various analyses to characterize their dynamical behaviors in different functional states. Penicillin G and its ring opening product serve as common ligands for these four proteins of interest. The dynamic analyses of overall structures, the active sites with penicillin G, and three catalytically important residues commonly shared by all four proteins reveal unexpected cross similarities between Class A β-lactamases and PBPs. These findings shed light on both the hidden relations among dynamical behaviors of these proteins and the functional and evolutionary relations among class A β-lactamases and PBPs.

## 1. Introduction

*β*-Lactam antibiotics have been used to treat bacterial infections since 1942. Antibiotics can interfere with the cross-linking in cell-wall biosynthesis, inhibiting cell wall growth and thus killing bacteria. As a mechanism of resistance for survival, bacteria produce *β*-lactamases to inactivate *β*-lactam antibiotics. The bacterial resistance to β-lactam antibiotic is an urgent and critical threat to global health. The main resistance mechanism involves antibiotic hydrolysis by *β*-lactamases through acylation and de-acylation catalytic cycles. β-Lactamases can be classified into four sub-groups (Classes A, B, C and D) based on their amino acid sequences and substrates [1]. Classes A, C and D are serine-based *β*-lactamases and class B are zinc-based *β*-lactamases. Among the four sub-groups, class A *β*-lactamases have a wide range of substrates and can spread via horizontal transfer, therefore posing a serious threat to public health [2] and have been widely studied [3].

**Figure 1:**
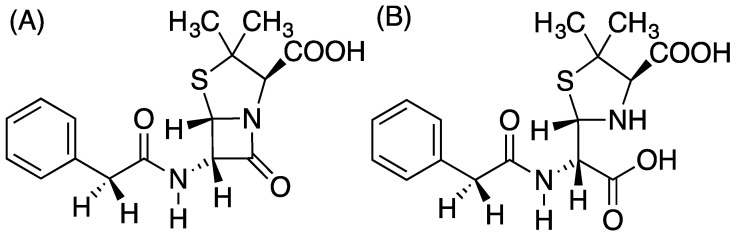
(**A**) Benzyl penicillin, (**B**) the hydrolysis product of benzyl penicillin.

Penicillin is the first commonly used *β*-lactam antibiotic. TEM-1, belonging to the Class A *β*-lactamases, can hydrolyze penicillin with high efficiency. TEM-1 has two conserved domains (α/β and α) around its active site. The structure of TEM-1 binding with benzyl penicillin (penicillin G) as substrate (PDB ID: 1FQG) at 1.7 Å resolution was reported in 1992 [4]. In the reported crystal structure, the penicillin G has a covalent bond with Ser70 as an intermediate structure. The catalytic mechanism of TEM-1 against penicillin was proposed to involve acylation and de-acylation steps (Figure 1). In the acylation step, the *β*-lactam ring of penicillin is attacked by the TEM-1 Ser70 residue. In the de-acylation step, TEM-1 Glu166 residue acts as a general base in the attack on the substrate assisted by a water molecule.

TOHO-1 is another Class A *β*-lactamase, and has an efficient hydrolytic activity against penicillin. Among Class A *β*-lactamases, TEM and the extended spectrum *β*-lactamases (ESBLs) CTX-M exhibit highly variable substrate profiles. TOHO-1 belongs to the ESBL CTX-M type enzymes and exhibits about 40% identity with the TEM families. The acyl-intermediate structure of TOHO-1 with penicillin G was reported in 2002 [5]. Like other *β*-lactamases, TOHO-1 has two highly conserved domains (α/β and α) around its active site. Ser70 at the active site of TOHO-1 is also critical for the hydrolysis of the penicillin molecule. The structure TOHO-1 apo forms with triple mutants was solved using neutron diffraction [6]. The Glu166 residue was proposed to act as a general base in the acylation reactions of TOHO-1 [7]. The catalytic mechanism of TOHO-1 against cefotaxime was investigated using both neutron and high-resolution X-ray diffraction. The study further emphasized the role of Lys73 in the acylation mechanism [8]. Additional studies also related TOHO-1 catalytic mechanism with the functions of active site residues [9,10,11].

Many reported structural evidences show that *β*-lactamases were evolved from cell wall biosynthetic enzymes, which are referred to as penicillin-binding proteins (PBPs) [12]. PBPs have a high sequence homology to class A *β*-lactamases. For example, the protein PBP-A shares a typical catalytic cavity and an overall fold with Class A *β*-lactamases, and 28% sequence similarities with *β*-lactamases on average [13]. Comparing the structure of PBP-A with Class A *β*-lactamases, PBP-A has a six residues deletion on the conserved Ω-loop, and there is no residue corresponding to Glu166 in TEM-1 as a general base in hydrolysis mechanism.

D-Alanyl-D-alanine transpeptidase (DD-transpeptidase), which was discovered in *Streptomyces* sp. R61 and classified as a PBP, has low sequence similarity compared to class A *β*-lactamases. DD-transpeptidase is a main target of penicillin and was proposed to share the same ancestor with *β*-lactamases [14]. Similar to TEM-1 and TOHO-1, DD-transpeptidase also has acylation and de-acylation catalytic steps for hydrolyzing penicillin. One main difference between *β*-lactamases (TEM-1 and TOHO-1) and DD-transpeptidase is that the de-acylation step reaction rate of DD-transpeptidase is extremely slow. Therefore, DD-transpeptidase can become effectively trapped in the acylated state. The crystal structure of DD-transpeptidase with penicillin G as a substrate was solved in 2004 and used to compare with the DD-transpeptidase complex with a peptidoglycan-mimetic *β*-lactam [15]. The structures and kinetic data from this study support the hypothesis that peptidoglycan-mimetic side-chains can improve the *β*-lactam inhibition activity [15]. In addition, Thr299 was identified as a highly conserved residue in the active site of DD-transpeptidase [16].

It was proposed that the majority of Class A *β*-lactamases and the present DD-transpeptidase were evolved from a same ancestor, most likely a DD-transpeptidase because of the similarity of substrate profiles, overall folds and the functional groups of the active site [17]. The ligand similarity could be used to cluster proteins with low sequence similarity. For example, a network-based model reported by Cheng et al. analyzes the interaction between proteins and ligands without structural information [18]. *β*-Lactamases and PBPs haven been studied through a ligand centric network model as well [19].

PBPs could undergo acylation reaction with penicillin G. PBP-A was identified as a member of a new family in PBPs due to a significant sequence similarity to class A *β*-lactamases. The crystal structure of PBP-A in apo state and acylated with penicillin G intermediate are both available from a study to evolve PBP-A into *β*-lactamase. In this study, PBP-A was compared with TEM-1 using structural alignment and hydrogen bond networks analysis [13]. Residue Glu166 was introduced to the shorter Ω-Loop of PBP-A, and a 90-fold increase in de-acylation rate was obtained. However, the sequence of PBP-A was not homologous with DD-transpeptidase [20].

Many computational methods, including molecular dynamic (MD) simulations and hybrid quantum mechanical and molecular mechanical (QM/MM) calculations were used to characterize the conformational changes and elucidate the catalytic mechanisms of protein structures [21]. The catalytic mechanism of DD-transpeptidase against cephalothin was studied using QM/MM method [22]. Tyr159 in DD-transpeptidase was proposed to carry functions different from the Tyr150 in *β*-lactamase as a general base. *β*-Lactamases have a rapid de-acylation step compared with DD-transpeptidase [23]. MD simulations could provide detailed dynamical insight into protein functions. Markov state model (MSM) is an effective method to model the kinetic information based on MD simulations [24,25].

In one of our previous studies, the dynamical properties of TEM-1 in different functional states, including complexes binding with penicillin G and its de-acylation product, respectively, and the apo state were characterized through MD simulations and machine learning methods [26]. The key residues for TEM-1 dynamics in different functional states were identified using machine learning methods.

In the current study, TEM-1, TOHO-1, PBP-A and DD-transpeptidase as structurally and functionally related proteins are subjected to extensive MD simulations and analyses. Despite the low sequence and structural homology, all four proteins could hydrolyze penicillin G, and are coupled with the evolution from ancient PBPs to *β*-lactamases. Detailed analyses were carried out using the hidden Markov state model (HMM) based on the overall enzyme structures and MSMs based on the active site binding with penicillin G to shed light onto the evolutionary relations among these four proteins.

## 2. Materials and Methods

**Figure 2:**
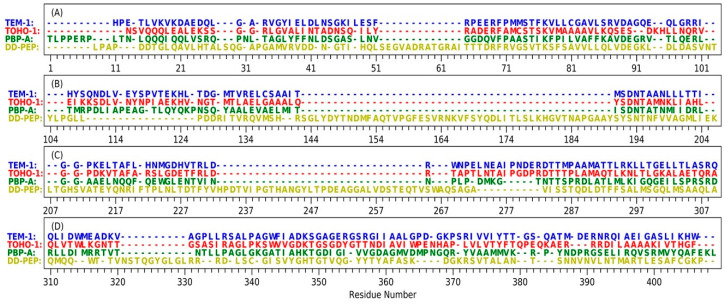
The sequence of four proteins including TEM-1, TOHO-1, PBP-A and DD-transpeptidase with STAMP structural alignment: (**A**) residue indices are from 1 to 103; (**B**) residue indices are from 104 to 206; (**C**) residue indices are from 207 to 309; (**D**) residue indices are from 310 to 409. TEM-1 residues are listed in blue text, TOHO-1 residues are listed in red text, PBP-A residues are listed in green text, DD-transpeptidase residues are listed in yellow text and DD-PEP represents DD-transpeptidase.

The initial structures of TEM-1, TOHO-1, Penicillin-Binding Protein (PBP-A) and DD-transpeptidase were obtained from the Protein Data Bank (PDB). Their PDB IDs are 1FQG [4], 1IYQ [5], 2J8Y [13] and 1PWC [15], respectively. All four selected crystal structures are in acylated state with covalent bonds to penicillin G intermediates. For each protein, three states were constructed: the apo state (a protein alone without ligand), reactant state (a protein binding with penicillin G, Figure 2A), and product state (a protein binding with the hydrolyzed product of penicillin G Figure 2B). It should be noted that no crystal structure for these enzymes in the reactant or product state is available in PDB. 

### 2.1. Molecular Dynamic Simulations 

The CHARMM36 force field was used to describe the selected enzymes [27]. CHARMM General Force Fields (CGenFF) [28,29] for penicillin G and its de-acylation product were generated using the online server ParamChem (https://cgenff.paramchem.org/). All simulation systems were solvated in water box using a TIP3P water model [30,31] with the addition of sodium and chloride ions to balance the charge and reproduce typical physiological ion concentrations. Initially, the simulation systems were subjected to 5000 steps of the steepest descent energy minimization and the adopted basis Newton-Raphson (ABNR) method with the gradient tolerance 0.02 kcal/mol·Å. Then, 10 ns of isothermal-isobaric ensemble (NPT) MD simulation was carried out for four proteins in each state. Subsequently, 1050 ns NVT ensemble MD simulations at 300 K were conducted. The first 50 ns simulations were discarded as equilibration and the following 1 μs was used for further analysis. The time step for MD simulations is 2 fs, and simulation trajectories were saved every 1000 steps (2 ps). All the bonds associated with hydrogen atoms were fixed during the simulation using SHAKE method [32]. Periodic boundary condition was used in all simulations, and electrostatic interactions were calculated using the particle mesh Ewald (PME) method [33]. All structural preparation and simulations were constructed using CHARMM simulation package version 41b1 with the support of GPU calculations based on OpenMM [34,35,36].

### 2.2. Root-Mean-Square Fluct+uation (RMSF) 

RMSF is a parameter to evaluate the fluctuation of conformation for each snapshot of the simulation from the averaged structures:

(1) RMSFi=[1T∑t=1T|ri(t)−ri¯|2]12

### 2.3. Principal Component Analysis (PCA)

PCA is a widely used dimensionality reduction method for molecular dynamics simulations [37,38]. It could be used to extract the dominant modes of the motion from a trajectory of molecular dynamic simulation. The normal modes for PCA were obtained through diagonalizing the correlation matrix of the atomic position in one trajectory. The correlation matrix element is calculated as:

(2) $$C_{ij}=\;\frac{c_{ij}}{\sqrt{c_{ii}c_{jj}}}=\;\frac{\langle r_{i}r_{j}\rangle -\langle r_{i}\rangle \langle r_{j}\rangle }{\sqrt{\left[\left(\langle r_{i}^{2}\rangle -\langle r_{i}\rangle {}^{2}\right)\left(\langle r_{j}^{2}\rangle -\langle r_{j}\rangle {}^{2}\right)\right]}}$$

where $C_{ij}$ is the variable of correlation matrix between atoms i and j.

### 2.4. Configurational Entropy

Entropy is estimated for the simulations using quasi-harmonic approximations based on MD simulations. Quasi-harmonic analysis is calculated by the inversion of the cross-correlation matrix C:

(3) $$F_{ij}=k_{B}T{\left[C^{-1}\right]}_{ij}$$

$F_{ij}$ is the element of the force constant matrix *F* describing the quasi-harmonic potential [39], $k_{B}$ is the Boltzmann constant and *T* is the temperature. The configurational entropy $S_{config}$ of the system can be calculated by the vibrational frequency *γ_i_* of the molecule with N atoms:

(4) $$S_{config}=k_{B}\overset{3N-6}{\underset{i}{\sum }}\frac{h\gamma_{i}/k_{B}T}{e^{h\gamma_{i}/k_{B}T}-1}-\ln\left(1-e^{-h\gamma_{i}/k_{B}T}\right)\;$$

where *h* is the Planck constant. The vibrational frequency *γ_i_* in the quasi-harmonic model *i* of a molecule can be calculated as the solution of the secular equation for angular frequency *ω*:

(5) $$\det\left(F-\omega^{2}M\right)=0$$

where M is the mass matrix of the molecule [37].

### 2.5. Time-Structure-Based Independent Component Analysis (t-ICA)

t-ICA [40,41,42,43] method was *n*-dimensional times series, t-ICA is performed by solving generalized eigenvalue problem:

(6) $$\bar{C}F=CKF$$

where $K=diag\left(k_{1},\ldots ,\;k_{n}\right)$ and $F=\left(f_{1},\ldots ,\;f_{n}\right)$ are the eigenvalue and eigenvector matrices, respectively. *C* is the covariance matrix, and $\bar{C}$ is the time-lagged covariance matrix at lagged time τ, which are defined as:

(7) $$C=\langle {\left(x\left(t\right)-\langle x\left(t\right)\rangle \right)}^{t}\left(x\left(t\right)-\langle x\left(t\right)\rangle \right)\rangle$$

(8) $$\bar{C}=\langle {\left(x\left(t\right)-\langle x\left(t\right)\rangle \right)}^{t}\left(x\left(t+\tau \right)-\langle x\left(t\right)\rangle \right)\rangle$$

Generally, the time-lagged covariance matrix is asymmetric. Avoiding complex numbers in eigenvectors and eigenvalues, a symmetrized time-lagged covariance matrix is given by 12(C¯+C¯t), which is under an assumption of time reversibility of a trajectory. The projected trajectories:

(9) a(t)= (a1(t), …,an(t))t=Fx(t)t

The independent component vectors obtained from t-ICA are uncorrelated and have the maximum autocorrelation value.

### 2.6. Markov State Models (MSMs)

A MSM [44,45] is used to reduce the complexity of the MD simulations by dividing the phase space into discrete microstates. The discrete microstates are generated by *k*-means clustering method. Consequently, the transition matrix could be computed, and the element in matrix $T_{ij}$ represents the probability of a microstate starting from microstate i, being transferred to microstate j after the lag time, τ. The dynamics of one system can be decomposed into independent processes represented by the eigenvectors of matrix T. The time scales of the process are computed from the eigenvalues, $\lambda_{i}$, of matrix T as:

(10) $$t_{i}=\;-\frac{\tau }{\ln\left|\lambda_{i}\right|}$$

### 2.7. Hidden Markov Model (HMM) 

It was shown that all important mechanistic molecular quantities, both kinetic and thermodynamic, computed by a Markov state model (MSM) are also computable from HMM [25,46,47]. In HMM framework, the basic assumption is that the full phase-space dynamics are Markovian in thermodynamic equilibrium. The dynamics can be projected onto the discrete clusters whose discrete dynamics was observed, which can generate so-called Projected Markov Models (PMMs). If the dynamics are metastable with a number of *m* slow relaxation processes, and the processes can transfer to the next-faster processes within a separation of timescales, then the PMMs can be approximated by HMM with *m* hidden states. A maximum likelihood transition matrix could be estimated among hidden states by an adequate lag-time. The lag-time dependent on estimated relaxation timescales is plotted in Supplementary Information (SI), and the lag time used in analysis is selected at the convergence of timescale. Meanwhile, the probability of a microstate belonging to a certain hidden state is estimated.

### 2.8. Transition-Path Theory 

In order to build the transition pathways, two subsets of the state space corresponding to unbound structures (initial states) and bound complex (end states) are defined to investigate the transition processes. All the other states are intermediate states. The committor probability $q_{i}^{+}$ is defined as the probability at state *i*, in which the system will reach the end state next rather than the initial state. According to the definition, $q_{i}^{+}=0$ for all *i* in initial state and $q_{i}^{+}=1$ for all *i* in end state. The committor probability for all intermediate states *i* can be calculated by the following equation:

(11) $$-q_{i}^{+}+\;\underset{k\in \;I}{\sum }T_{ik}q_{k}^{+}=-\underset{k\in end\;states}{\sum }T_{ik}$$

The committor increases from an initial state to an end state. The effective flux $f_{ij}=\;\pi_{i}q_{i}^{-}T_{ij}q_{j}^{+}$, where $\pi_{i}$ is the stationary probability when the transition matrix $T_{\tau }$ is normalized. Here, $T_{\tau }$ has a single eigenvector and eigenvalue 1, since it is ergodic (within finite time any state can be reached from any other state), $q_{i}^{-}$ is the probability at state *i* and previously at an initial state. In equilibrium of a molecule, $q^{-}=1-q^{+}$. For any intermediate states pair i, j, both the $f_{ij}$ and $f_{ji}$ are positive. If the $f_{ij}^{+}$ is only considered, $f_{ij}^{+}=max\left\{0,\;f_{ij}-f_{ji}\;\right\}$. The total flux can be calculated by:

(12) $$F=\underset{i\;\in \;initial\;state}{\sum }\underset{j\;\notin initial\;state}{\sum }\pi_{i}T_{ij}q_{j}^{+}$$

The individual pathways $p_{i}$ connecting an initial state to an end state can be decomposed from the flux. In equilibrium, a flux can be nonuniquely decomposed into pathways from an initial state to an end state. The decomposition generates a set of pathways $p_{i}$ along $f_{i}$. The $f_{i}$ provides a relative probability when the set of pathways $p_{i}$ is considered [24,48]:

(13) pi=fi∑jfj

In the current study, the PCA of all α-carbon coordinates for four proteins in three states are analyzed by Bayesian HMM [46]. HMM is used to explore the conformational changes of overall structures of four proteins in each state. α-Carbons are backbone carbons, which represent the relative position with reference to other functional groups. Protein α-carbons have been used in many computational methods, such as principal component analysis and recurrence quantification analysis, to elucidate the essential dynamics and functions of proteins [49,50,51]. In this paper, α-carbon coordinates were subjected to HMM analysis to extract the overall dynamic motions of proteins in three states. To apply HMM, simulations were first grouped into microstates through clustering analysis. Then several macrostates could be identified with appropriate lag time and estimated transition probabilities. The lag-time and number of macrostates for each protein in different states are not unique (time scales dependent on lagged time listed in Supplementary Figures S1–S4). The top two tICs (lag-time 2 ns used for all four proteins) of pairwise distances among heavy atoms of residues in active site (residues are listed in Table 1) were carried out for each protein in different states. MSMs are constructed to elucidate the conformational changes of active site for each protein complex with penicillin G. Perron-cluster cluster analysis (PCCA) was applied to map microstates onto macrostates. The lag-time are selected depending on the implied timescale plots listed in Supplementary Figure S5. The lag time at the end of the x-axis is the adequate lag time (Supplementary Figures S1–S5) used in further construction. PyEMMA version 2.5.6 [25,52,53,54] was employed to build Markov state Models and Hidden Markov state Model. Other parameters besides lag time in the construction of models are set to their default values.

## 3. Results

### 3.1. Hidden Markov State Models Analysis of Overall Structures

The structures of TEM-1 (PDB ID: 1FQG), TOHO-1 (PDB ID: 1IYQ), PBP-A (PDB ID: 2J8Y) and DD-transpeptidase (PDB ID: 1PWC) were aligned for the comparisons in structure and sequence (Figure 2). All four proteins are subjected to a structural alignment by MultiSeq tool in VMD under STAMP structural alignment algorithm [55,56]. TEM-1 and TOHO-1 both belong to class A β-lactamases and have a very high sequence similarity. PBP-A and DD-transpeptidase belong to the penicillin-binding proteins (PBPs). Although PBPs were reported to share low sequence similarity with β-lactamases [13], PBP-A does show high homology level with TEM-1 and TOHO-1. Comparing to the other three proteins, DD-transpeptidase has several long insertions in its structure. The longest sequence insertion of DD-transpeptidase (residue number 143 to 190 in Figure 1B) was close to the Ω-loop of TEM-1 (residue number 191 to 200 in Figure 1B). The residue numbers listed in the Figure 2 are based on the STAMP structural alignment and do not represent the real residue ordered in the PDB structures.

**Scheme 1:**
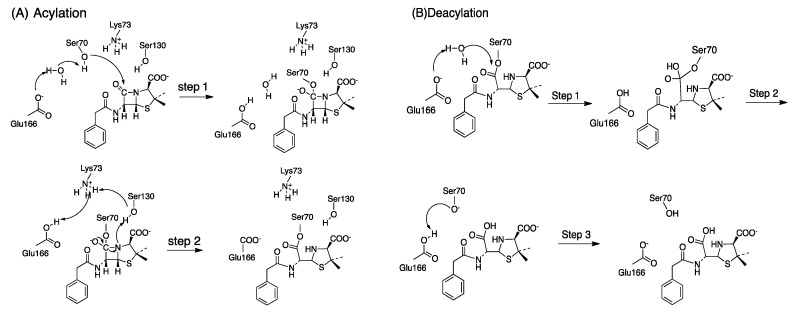
(**A**) The acylation mechanism of Class A β-lactamases, Step 1: The nucleophilic attack and formation of the tetrahedral intermediate; Step 2: Formation of the acylenzyme from the tetrahedral intermediate (**B**) The deacylation mechanism of Class A β-lactamases, Step 1: The nucleophilic attack by a water molecule and formation of the tetrahedral intermediate; Step 2: The bond-breaking between Ser70-O of the enzyme and the β-latam carbonyl carbon atom; Step 3: Hydrogen atom transfers from Glu166 to Ser70-O and the regeneration of protonation state of the enzyme.

The acylation mechanism of Class A β-lactamase is divided into two steps, shown in Scheme 1A [57]. In the first step, a proton is abstracted from Ser70 and another proton is transferred to Glu166 via water as a bridge. The nucleophilic (deprotonated Ser70) attacks the carbonyl group of the β-lactam ring. In this step, the tetrahedral intermediate structure is formed. Next, the cleavage of the β-lactam bond is accompanied by Ser130, Lys73 is neural, and Glu166 remains protonated. Then, a proton transfers from Glu166 to Lys73, with the formation of the acyl-enzyme.

The deacylation mechanism of Class A β-lactamase is divided into three steps, shown in Scheme 1B [58]. In the first step, the nucleophilic attack catalyzed by the hydrolytic water molecule occurs on the carbonyl carbon of the acyl-enzyme. And the Glu166 plays as a general base accepting the proton from the water molecule. In the second step, the bond between Ser70-O and the β-latam carbonyl carbon atom is broken. In the third step, the protonation state of the protein is regenerated through the hydrogen transfer from the Glu166 carboxylate to the Ser70-O.

**Figure 3:**
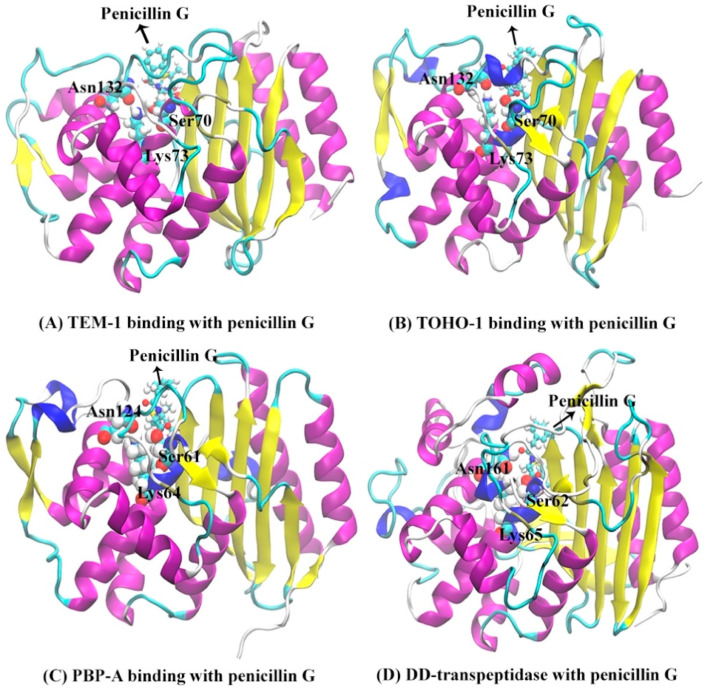
The structures of proteins binding with penicillin G molecule (red sticks): (**A**) TEM-1; (**B**) TOHO-1; (**C**) PBP-A; (**D**) DD-transpeptidase. The binding pockets are identified around three active site residues represented by balls (Ser70, Lys73 and Asn132 for TEM-1; Ser70, Lys73 and Asn132 for TOHO-1; Ser61, Lys64 and Asn124 for PBP-A; Ser62, Lys65 and Asn161 for DD-transpeptidase), penicillin G is illustrated as balls and sticks.

The secondary structures of TEM-1, TOHO-1, PBP-A and DD-transpeptidase are illustrated in Figure 3 with the penicillin G molecule shown as sticks and balls. There are three unique active site residues shared by all four proteins. Through the comparison among the TOHO-1 (1IYQ) [5] and TEM-1 (1FQG) [4] active sites in acyl-intermediate structures, PBP-A (2J8Y) [13] active site binding with penicillin G, and DD-transpeptidase (1PWC) [15] active site binding with penicillin G, three residues are shared among all four proteins. These common active site residues are Ser70, Lys73 and Asn132 in TEM-1, Ser70, Lys73 and Asn132 in TOHO-1, Ser61, Lys64 and Asn124 in PBP-A, and Ser62, Lys65 and Asn161 in DD-transpeptidase. These three common active residues are illustrated in Figure 3.

The Hidden Markov state Model (HMM) analyses were carried for TEM-1 in apo, reactant and product states based on the principal component analysis (PCA) of all α carbon coordinates in each state. In each state of TEM-1, the saved snapshots from simulations are projected onto the top two main vectors referred to as the principal component 1 (PC1) and principal component 2 (PC2) space. The projected snapshots are consequently subjected to the *k*-means clustering analysis and divided into 200 microstates on the PC1/PC2 surface. Evaluations of the implied timescale show convergences at lag time 160 ps for the apo state, 200 ps for the reactant state, and 180 ps for the product state (Supplementary Figure S1). The HMM was applied to construct metastable macrostates from the clustered microstates using appropriate lag time for each state.

**Figure 4:**
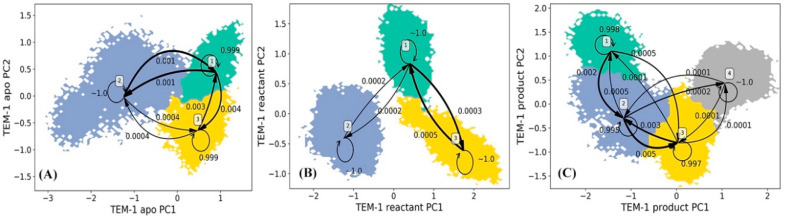
Hidden Markov state model (HMM) of TEM-1 apo (**A**), reactant (**B**), and product (**C**) states. The macrostates resulted from the HMM are based on the 200 microstates generated using *k*-means clustering analysis of TEM-1 simulations in each state. The simulations of each TEM-1 state are projected onto two main vectors referred to as the principal component 1 (PC1) and principal component 2 (PC2) from the principal component analysis (PCA) of all α carbon coordinates in each state simulations. The transition probabilities among macrostates in HMM are also listed. Overall, the probability to remain in the macrostate is overwhelmingly higher than transferring to any other macrostates.

The HMM analyses of TEM-1 result in three, three, and four metastable macrostates for the apo, reactant and product states, respectively (Figure 4). The metastable macrostates are illustrated in different colors for comparison purpose. In the three states of TEM-1, the macrostates distributed in a similar area on the PCA surface are in the same color, suggesting that these macrostates share a similar dynamic behavior. The transition probabilities among the macrostates for each TEM-1 state were calculated using HMM. For each macrostate, the probability to remain in the current state is overwhelmingly higher than the probability transferring to any other states. Therefore, each macrostate represents a free energy minimum on the surface with kinetic barriers preventing transformations to other macrostates. The simulation of TEM-1 apo state comprises three macrostates (Figure 4A). The macrostate 2 with the largest coverage on the PCA surface is the dominant state among three macrostates. The three macrostates in TEM-1 reactant state (Figure 4B) have an arrangement similar to the apo state, suggesting a similar dynamical behavior for the TEM-1 in these two functional states. Interestingly, the interconversion between macrostates 2 and 3 is extremely unlikely in the reactant state (Figure 4B). This demonstrates the significant impact from the binding with penicillin G on the dynamical properties of TEM-1. The TEM-1 product state simulations were divided into four macrostates (Figure 4C). The distribution of these four macrostates on the PCA surface is significantly different from the apo and reactant states. The interconversion between macrostates 1 and 4 in the product state is extremely unlikely (Figure 4C).

**Figure 5:**
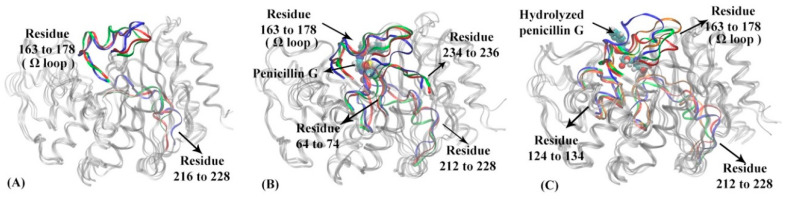
The representative structures of macrostates in three functional states of TEM-1: (**A**) apo state, (**B**) reactant state, (**C**) product state. In TEM-1 apo state (**A**): residue 163 to 178 (Ω loop), residue 216 to 228 are highlighted in macrostate 1 (blue), macrostate 2 (red), macrostate 3 (green). In TEM-1 reactant state (**B**): residues 64 to 74, residues 163 to 178 (Ω loop), residues 212 to 228 and residues 234 to 236 are highlighted in macrostate 1 (blue), macrostate 2 (red), macrostate 3 (green). The penicillin G ligand is illustrated as space filling model in the binding pocket. In TEM-1 product state (**C**): residues 124 to 134, residues 163 to 178 (Ω loop) and residues 212 to 228 are highlighted in macrostate 1 (blue), macrostate 2 (red), macrostate 3 (green), and macrostate 4 (orange). The hydrolyzed penicillin G (product state) is illustrated as space filling model in the binding pocket.

To further understand the dynamical behaviors of TEM-1 in different functional states, the representative structures for each macrostate in apo, reactant and product states of TEM-1 are illustrated in Figure 5. The RMSFs for each macrostate of TEM-1 in three states are plotted in Supplementary Figure S10 to distinguish the key conformational change. Key secondary structures with significantly different RMSF values in three functional states are highlighted in different colors (Figure 5). Penicillin G and its hydrolyzed product as ligands in the reactant and product states are illustrated in space filling mode. Residues 163 to 178 located around the active site in the TEM-1 structure is referred to as Ω loop, which is a critical functional group in the catalytic process of TEM-1 with penicillin G.

The structural differences among three macrostates in the TEM-1 apo state mainly stem from the Ω loop and residues 216 to 228 (Figure 5A). The residues 216 through 228 also form a loop-like structure, which is located at the distal end of the active site and displays certain flexibility in the apo state. 

In the reactant state, the Ω loop and residues 216 to 228, 64 to 74, 234 to 236, and 211 to 215 display significant differences among three macrostates (Figure 5B). Compared to the apo state, the binding with the penicillin G ligand changes the distribution of Ω loop in the reactant state, in favor of conformations closer to the ligand. In addition, the binding with the ligand diminishes the flexibility of loop region residues 212 to 228. Residues 64 to 74 and 234 to 236 as two loops adjacent to the active site, however, show a slightly higher flexibility than in the apo state. Some key catalytic residues for TEM-1, including Ser70, Lys73, Ser235, belong to these regions.

The representative structures of four macrostates in TEM-1 product state are illustrated in Figure 5C. Surprisingly, the Ω loop displays much higher flexibility than both the apo and reactant states. But a loop region of residues 212 to 228 displays conformations similar to the reactant state. Different from the apo and reactant states, another loop region of residues 124 to 134 displays significant conformational changes of TEM-1 in the product state. Interestingly, the residues 130, 131, 132 in this loop is important functional residues in catalytic mechanism [59,60].

**Figure 6:**
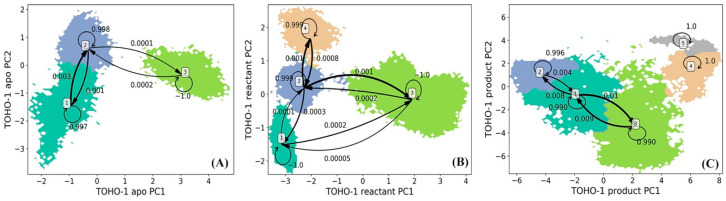
Hidden Markov state model (HMM) of TOHO-1 (**A**) apo, (**B**) reactant, and (**C**) product states. The macrostates resulted from the HMM are based on the 200 microstates generated using *k*-means clustering analysis of TOHO-1 simulations in each state. The simulations of each TOHO-1 state are projected onto two main vectors referred to as the principal component 1 (PC1) and principal component 2 (PC2) from the principal component analysis (PCA) of all α carbon coordinates in each state simulations. The transition probabilities among macrostates in HMM are also listed.

The simulations of TOHO-1 in apo, reactant and product states are also projected onto the surfaces formed by top two vectors (PC1 and PC2) from the PCA using all α carbon coordinates for each state. The projected snapshots were consequently subjected to the *k*-means clustering analysis and divided into 200 microstates based on structural differences. HMM was then applied on the microstates to generate macrostates. The lag time used in the construction of HMM for TOHO-1 is 160 ps for the apo state, 160 ps for the reactant state, 180 ps for the product state as shown in Supplementary Figure S2A–C. The HMM analysis of TOHO-1 resulted into three, four, and five macrostates for the apo, reactant and product states, respectively (Figure 6). The transition probabilities among macrostates in each TOHO-1 functional state is also estimated using HMM and labeled (Figure 6). The probability of each macrostate remaining in itself is much higher than the probabilities of transferring to other macrostates, making each macrostate as a free energy minimum.

**Figure 7:**
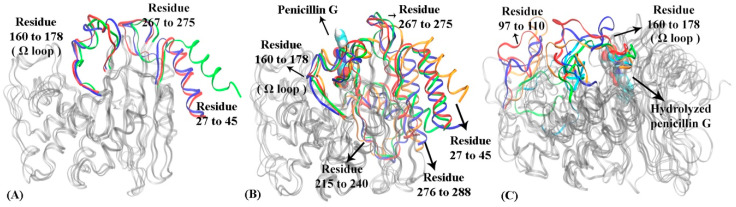
The representative structures of macrostates in three functional states of TOHO-1: (**A**) apo state, (**B**) reactant state, (**C**) product state. In TOHO-1 apo state (**A**) N-terminus helix (residues 27 to 45), a loop of residues 267 to 255 and Ω loop (residues 160 to 178) are highlighted in macrostate 1 (blue), macrostate 2 (red), and macrostate 3 (green). In TOHO-1 reactant state (**B**) N-terminus helix (residues 27 to 45), Ω loop (residues 160 to 178), loop of residues 215 to 240, loop of residues 267 to 275 and loop of residues 275 to 288 are highlighted in macrostate 1 (blue), macrostate 2 (red), macrostate 3 (green) and macrostate 4 (orange); the penicillin G is illustrated in space filling mode at the binding pocket. In TOHO-1 product state (**C**) loop of residues 97 to 110 and Ω loop (residues 160 to 178) are highlighted in macrostate 1 (blue), macrostate 2 (red), macrostate 3 (green), macrostate 4 (orange) and macrostate 5 (cyan). The hydrolyzed penicillin G (product state) is illustrated in the space filling mode.

The HMM analysis of TOHO-1 apo state resulted into three macrostates (Figure 6A). The macrostates 1 and 2 are adjacent to each other with relatively high transition probabilities among them. The representative structures of three macrostates are illustrated in Figure 7A and the key conformational changes are highlighted in blue, red and green colors for macrostates 1, 2 and 3, respectively. Surprisingly, the TOHO-1 apo state does not show a significant flexibility. The Ω loop (residues 160 to 178) displays a limited flexibility. The main conformational changes among three macrostates lie in the helix of N-terminus (residues 27 to 45), which swings away from the protein in the macrostate 3 (highlighted in green).

The distribution of four macrostates in TOHO-1 reactant state (Figure 6B) closely resembles the distribution of the macrostates in TOHO-1 apo state (Figure 6A). Macrostates 1, 2, and 4 in the TOHO-1 reactant state lie in the left-hand side of the PC1-PC2 surface, covering the area corresponding to the macrostates 1 and 2 in the TOHO-1 apo state. The distributions of macrostates 1, 2, and 4 of TOHO-1 reactant state show little overlapping between adjacent states 1 and 2 as well as adjacent states 2 and 4. The representative structures of macrostates 1 to 4 are illustrated in Figure 7B. The secondary structures with significant conformational changes among different macrostates are highlighted in blue (macrostate 1), red (macrostate 2), green (macrostate 3) and orange (macrostate 4). It is interesting that the helix at the N-terminus (residues 27 to 45) in the reactant state shows more flexibility than the apo state. The Ω loop shows the comparable flexibility to the apo state, but there is a loop region (residue 267 to 275) close to the ligand displaying significant conformational changes among different macrostates. In addition, the loop comprising residues 215 to 240 and helix comprising residues 276 to 288 also display higher flexibilities in the reactant state than in the apo state. Overall, the binding with penicillin G seems to increase the overall flexibility and conformational distribution of TOHO-1.

The distribution of five macrostates in TOHO-1 product state (Figure 6C) is dramatically different from the apo and reactant states. The distributions of macrostates 1, 2 and 3 are close to each other and cover the majority of the surface. The transition probabilities between states pairs 1 and 3, 1 and 2 are rather high. Transitions to and from states 4 or 5 are rather rare, and do not lead to meaningful transition probabilities associated with either of these two states, suggesting that these two states are rather isolated in the product state. The representative structures of these five macrostates are illustrated in Figure 7C. Interestingly, the binding mode of the hydrolysis product of penicillin G is quite different from the binding mode in the reactant state, and leads to a different dynamical behavior of the protein. The Ω loop shows a significant flexibility comparing to the apo and reactant states. This is actually similar to the case of TEM-1. Another loop region of residues 97 to 110 of TOHO-1 also shows a higher flexibility than in both apo and reactant states of TOHO-1. Both of these two loops are away from the ligand of product state. On the other hand, the N-terminus (residues 27 to 45) is more rigid in the product state than in the apo and reactant states. In both TEM-1 and TOHO-1 case, the binding with hydrolysis product of penicillin G leads to the dynamical behavior of the protein dramatically different from both the apo and reactant states. These findings indicate the importance of dynamical behavior in different functional states of β-lactamases. It is also worth to point out that the dynamical behaviors of both TEM-1 and TOHO-1 in product state are significantly different from the apo and reactant states. This may suggest that the key differences in dynamical behaviors of β-lactamases in different functional states are important for their catalytic functions.

**Figure 8:**
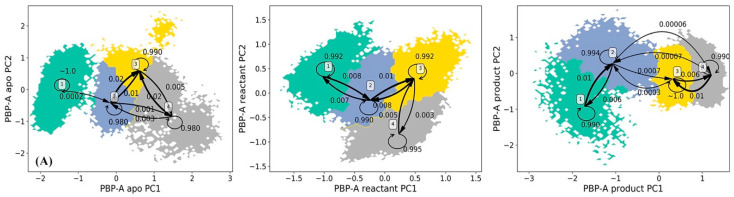
Hidden Markov state model (HMM) of PBP-A (**A**) apo, (**B**) reactant, and (**C**) product states. The macrostates resulted from the HMM are based on the 200 microstates generated using *k*-means clustering analysis of PBP-A simulations in each state. The simulations of each PBP-A state are projected onto two main vectors referred to as the principal component 1 (PC1) and principal component 2 (PC2) from the principal component analysis (PCA) of all α carbon coordinates in each state simulations. The transition probabilities among macrostates in HMM are also listed.

**Figure 9:**
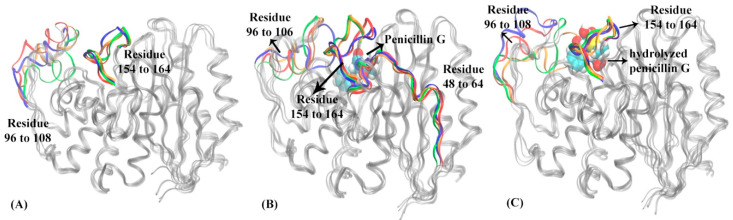
The representative structures of macrostates in three functional states of PBP-A: (**A**) apo state, (**B**) reactant state, (**C**) product state. In PBP-A apo state (**A**): loop of residues 96 to 108 and Ω loop (residues 154 to 164) are highlighted in macrostate 1 (blue), macrostate 2 (red), macrostate 3 (green) and macrostate 4 (orange). In PBP-A reactant state (**B**): residues 48 to 64, loop of residues 96 to 106, and Ω loop of residues 154 to 164 are highlighted in macrostate 1 (blue), macrostate 2 (red), macrostate 3 (green) and macrostate 4 (orange). The penicillin G is illustrated in space filling mode in the binding pocket. In PBP-A product state (**C**): loop of residues 96 to 108 and Ω loop of residues 154 to 164 are highlighted in macrostate 1 (blue), macrostate 2 (red), macrostate 3 (green), macrostate 4 (orange). The hydrolyzed penicillin G (product state) is illustrated in space filling mode in the binding pocket. The hydrolyzed penicillin G (product state) is represented in the space filling mode.

The HMM analysis was carried out for the simulations of PBP-A in apo, reactant, and product states similar to TEM-1 and TOHO-1, and lead to four macrostates in each functional state of PBP-A (Figure 8). The lag times used for PBP-A three states is are 180 ps for the apo state, 180 ps for the reactant state, 160 ps for the product state as shown in Supplementary Figure S3. In the apo state of PBP-A, macrostates 2, 3, and 4 are adjacent to each other, with significant transition probabilities among them. The macrostate 1 is separated from the macrostates 2, 3, and 4, and is connected to macrostate 2 with a detectable transition probability. The representative structures of four macrostates in PBP-A apo states are illustrated in Figure 9A. Among the macrostates of apo state, the Ω loop (residues 154 to 164) does not show significant conformational changes. But a loop region of residues 96 to 108 at the active site shows significant conformational changes. Despite the overall structural difference, the structural alignment between PBP-A and TEM-1 shows that the Ω loop in PBP-A (residues 152 to 166) aligns well to the Ω loop of TEM-1.

All four macrostates in the reactant state of PBP-A are clustered together with significant transition probabilities among them (Figure 8B). This indicates that the PBP-A bound with penicillin G in reactant state may not be flexible. The representative structures of four macrostates of PBP-A in the reactant state are illustrated in Figure 9B. The binding with the ligand diminishes the flexibility of overall structure of PBP-A, especially the loop region of residues 96 to 108. However, the Ω loop (residues 152 to 166) shows somewhat a higher flexibility in the reactant state than in the apo state. The residues 48 to 64 include a key active site residue Ser 61. Residues 48 to 64 have a relative high flexibility because of a flexible Ser61 and adjacent residues. Although the region does not seem to have high conformational changes in representative structures, the actual RMSFs of this region are higher than other regions as plotted in Supplementary Figure S17.

The four macrostates in the product state of PBP-A are also clustered together with significant transition probabilities among them (Figure 8C). This may suggest that the overall structural flexibility of PBP-A in this state is low. The representative structures of four macrostates in the product state are illustrated in Figure 9C. The Ω loop (residues 152 to 166) shows similar conformations to the apo state. Interestingly, the loop of residues 96 to 108 is more flexible than in both apo and reactant states.

**Figure 10:**
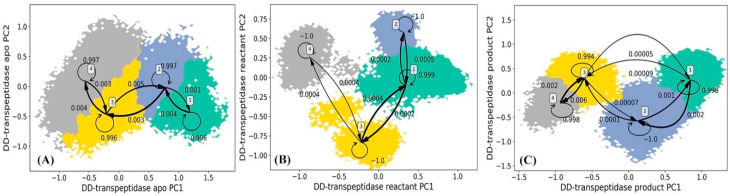
Hidden Markov state model (HMM) of DD-transpeptidase (**A**) apo, (**B**) reactant, and (**C**) product states. The macrostates resulted from the HMM are based on the 200 microstates generated using *k*-means clustering analysis of DD-transpeptidase simulations in each state. The simulations of each PBP-A state are projected onto two main vectors referred to as the principal component 1 (PC1) and principal component 2 (PC2) from the principal component analysis (PCA) of all α carbon coordinates in each state simulations. The transition probabilities among macrostates in HMM are also listed.

**Figure 11:**
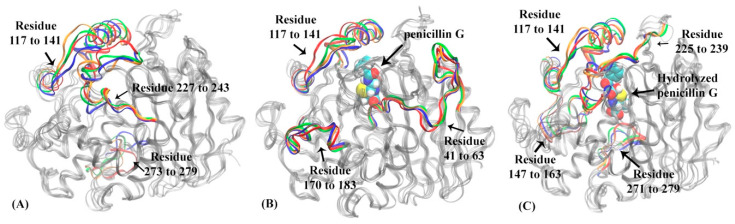
The representative structures of macrostates in three functional states of DD-transpeptidase: (**A**) apo state, (**B**) reactant state, (**C**) product state. In DD-transpeptidase apo state (**A**): residues 117 to 141, residues 227 to 243 and residues 273 to 279 are highlighted in macrostate 1 (blue), macrostate 2 (red), macrostate 3 (green) and macrostate 4 (orange). In DD-transpeptidase reactant state (**B**): residues 41 to 63, residues 117 to 141 and residues 170 to 183 are highlighted in macrostate 1 (blue), macrostate 2 (red), macrostate 3 (green) and macrostate 4 (orange). The penicillin G is represented in space filling mode in the binding pocket. In DD-transpeptidase product state (**C**): residues 117 to 141, residues 147 to 163, residues 225 to 239 and residues 271 to 279 are highlighted in macrostate 1 (blue), macrostate 2 (red), macrostate 3 (green), macrostate 4 (orange). The hydrolyzed penicillin G (product state) is illustrated in the space filling mode.

The HMM analysis was carried out for the simulations of DD-transpeptidase in the apo, reactant, and product states similar to the other three proteins, and lead to four macrostates in each functional state of PBP-A (Figure 10). The lag times used in the construction of HMM for DD-transpeptidase are 180 ps for the apo state, 160 ps for the reactant state, and 180 ps for the product state as shown in Supplementary Figure S4A–C. In the apo state of DD-transpeptidase, all four macrostates are adjacent to each other with significant transition probabilities among them (Figure 10A). Similar to the PBP-A analysis, this could indicate that the protein does not show a high flexibility in this state. The representative structures of four macrostates in the apo state are illustrated in Figure 11A. Residues 117 to 141 with mixed loop and helix, residues 227 to 243 (corresponding to the Ω loop in TEM-1 under structural alignment in Figure 2C), and the loop of residues 273 to 279 are highlighted with the significant conformational variance in overall DD-transpeptidase structure (Figure 11A). It should be noted that the residues 117 to 141 are located in an insertion of sequence (among residues 110 to 157 as shown in Figure 2B). Residues 273 to 279 are among another insertion of sequence (residues 275 to 284).

Comparing to the apo state, the four macrostates in the reactant state of DD-transpeptidase are separated from each other (Figure 10B). The representative structures of these macrostates in the reactant state are illustrated in Figure 11B, and do not show a significant difference from the apo state structures. Two loops (residues 41 to 63 and residues 170 to 183) and residues 117 to 141 (including mixed loop and helix) with significant conformational changes among four macrostates are highlighted. Residues 117 to 141 are in an insertion of sequence in DD-transpeptidase comparing to TEM-1, TOHO-1 and PBP-A structure. Residues 41 to 63 includes a key active site residue Ser62. A loop formed by residues 170 to 183 away from the active site shows a higher flexibility in the reactant state than in the apo state.

Four macrostates in the product state of DD-transpeptidase are rather close to each other on the PCA surface (Figure 10C). The distribution of these four states are similar to both the apo and reactant states, which indicates a similar dynamical behavior in three functional states of DD-transpeptidase. The representative structures of these macrostates are illustrated in Figure 11C. The flexibility displayed by the residues 117 to 141 is similar to those in the apo and reactant states. The other three loops region, residues 147 to 163, residues 225 to 239 and residues 271 to 279 with significant conformational changes do not show high flexibilities either. All the four loops are located around hydrolyzed product of penicillin G as a ligand.

### 3.2. Analysis of Active Site Structures Using Markov State Models

The above analyses strongly indicate that TEM-1 and TOHO-1 as two Class A *β*-lactamases share a similar dynamical behavior in different catalytic functional states, including binding with either reactant or product and the apo state as well. PBP-A and DD-transpeptidase, on the other hand, share a similar dynamical behavior in different catalytic functional states. All four proteins share a similar catalytic cavity. The active site residues of each protein are listed in Table 1. To further analyze and directly compare the dynamical behaviors of these proteins related to their catalytic activities, the active site of each protein combining with the reactant penicillin G are subjected to analysis using MSM based on t-ICA. The lag times of the MSM for four proteins are 3 ns as shown in Supplementary Figure S5. 

**Figure 12:**
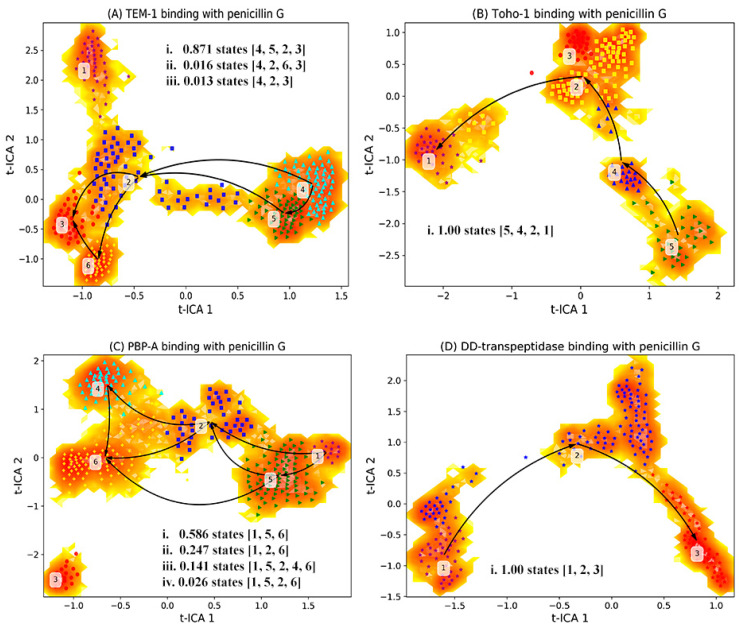
Markov state model analysis of reactant state simulations for (**A**) TEM-1, (**B**) TOHO-1, (**C**) PBP-A, (**D**) DD-transpeptidase. The simulations are subjected to t-ICA to identify the top two vectors (t-ICA 1 and t-ICA 2), upon which the simulations are projected. Based on the representative structures, different macrostates are identified as initial state (unbound state), intermediate states, end state (bound state) and trapped states. It should be noted that the trapped states are terminal states without being on the transition pathway connecting the bound and unbound states. The unbound states are those structures that are the most different from the bound structure, and are not expected to be a completely dissociated state between the active site and ligand.

The main focus is the dynamical process of binding between the protein active site and the ligand. During the simulations of all four proteins, the escaping of the ligand from the active site was observed. Only the simulations of reactant state for each protein were subjected to the analysis. Therefore, the binding process of the ligand from the active site in each protein could be characterized as macrostates generated from the MSM. The distribution of macrostates in the reactant state simulations of each protein are plotted in Figure 12. 

**Figure 13:**
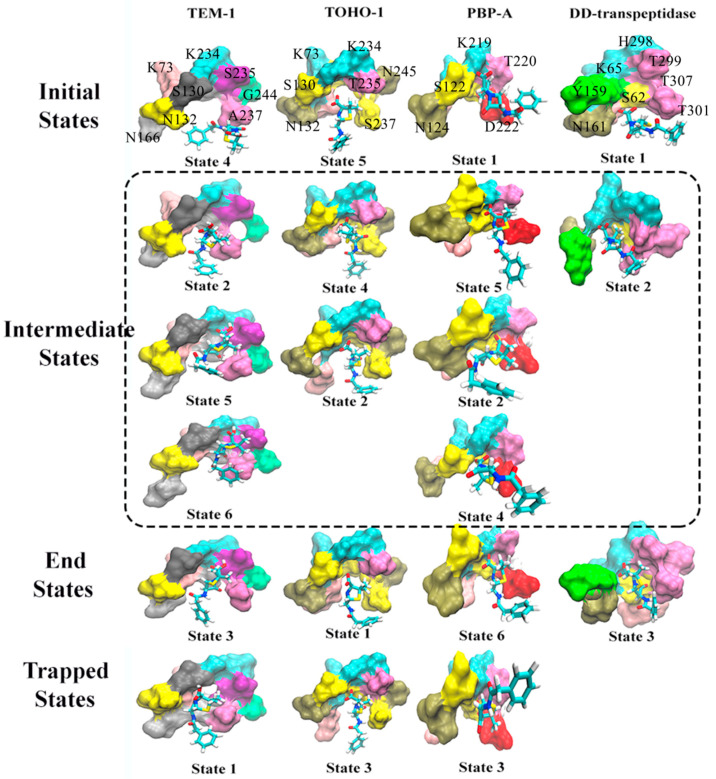
The representative structures of active site of TEM-1, TOHO-1, PBP-A and DD-transpeptidase with penicillin G as a ligand corresponding to the macrostates in Figure 12. The macrostates are divided into four states: initial state (unbound state), intermediate states, end state (bound state) and trapped states. The trapped states are those states that are terminal states without being on the transition pathway connecting the bound and unbound states. The unbound states are those structures that are the most different from the bound structure, and are not expected to be a completely dissociated state between the active site and ligand. The residues in each active site are illustrated as surface in different colors, and penicillin G molecule is illustrated in stick model. The residues in TEM-1 active site include S70, K73, S130, N132, N166, K234, S235, A237, G244; the residues in TOHO-1 active site include S70, K73, S130, N132, A166, K234, T235, S237, N245; the residues in PBP-A active site include S61, S64, S122, N124, L158, K219, T220, D222, G228; the residues in DD-transpeptidase active site include S62, K65, Y159, N161, E237, H298, T299, T301, T307. The residue names are labeled in the initial states, some residues hidden in the back are not labeled.

Such an analysis reveals dominant transition pathways of the system of interest. For example, states sequence [2,3,4,5] in Figure 12A represents a transition pathway 4→5→2→3 with probability 0.871, with state 4 is an initial state and state 3 is an end state. The representative structures of active site combining with reactant for macrostates generated from the MSM of trajectories are illustrated in Figure 13. These representative structures are divided into the initial state (unbound state), intermediate states, end state (bound state) and trapped states. The trapped states are those states that are terminal states without being on the transition pathway connecting the bound and unbound states. It should be noted that the unbound states are those structures that are the most different from the bound structure, and are not expected to be a completely dissociated state between the active site and ligand. The trapped states can be described as local minima on kinetic energy surface, separated by energy barrier from the main basin. Such trapped states are rarely visited, and are stable for significant amount of time if they are actually visited [61].

Based on the transition probabilities among these macrostates, one could identify the most probable transition pathways connecting the bound and unbound states. For example, the most probable transition pathway from the unbound state to intermediate state to the bound state for TEM-1 binding with penicillin G is macrostates 4→5→2→3 with transition probability as 0.871 (Figure 12A). The probability is calculated using the committor probability described in transition-pathway theory. The representative structures for macrostates 2–5 illustrated in Figure 13A demonstrate the conformational change of active site with the ligand throughout the binding process. There are other possible transition pathways, including 4→2→6→3 and 4→2→3, which bear much lower transition probabilities. In the macrostate 4 (initial state), the penicillin G resides at the cavity of TEM-1 with a weak interaction with Ala237. In the intermediate states, residue Asn132 in the macrostates 2, 5 and 6 moves opposite to the penicillin G and creates additional space for the ligand. This behavior of Asn132 was reported in an experimental paper [59]. In the six macrostates of TEM-1, residues Ala237 and Lys73 have significant conformational changes. Ala237 moves closer to the ligand. From the initial state to the end state, residue Lys73 gradually moves to the back side and closer to Ser70.

The most probable transition pathway of TOHO-1 binding with penicillin G is identified through MSM as macrostates 5→4→2→1 (Figure 12B). This transition pathway is illustrated in Figure 13. The trapped state 3 seems to be similar to the bound state. Overall, the active site of TOHO-1 reactant state does not show a high flexibility. Only Asn132 has a significant conformational change in this case. Similar to TEM-1, the binding pocket of TOHO-1 is broadened in the intermediate states (macrostates 2 and 4) by the rotation of sidechain of residue Asn132.

The macrostates distributions of active site with penicillin G in PBP-A cover a wide range of t-ICA surface constructed by the two main vectors. The significant transition probabilities among multiple macrostates lead to multiple transition pathways between the bound and unbound state (Figure 11C). The most probable transition pathway is 1→5→6. The second most probable transition pathway is 1→2→6. The representative structures are illustrated in Figure 13. Other transition pathways are less probable. It is interesting that the transition pathway 1→5→2→6 is less probable than the pathway as 1→5→2→4→6, because of the higher transition probability for transition of 2→4→6 than the one for transition of 2→6. The main conformational changes along the transition pathway are from Asn124 and Asp222, which correspond to Asn132 and Ala237 in TEM-1 active site. Residue Asn124 has a similar motion to provide space for the ligand in the intermediate states. However, residue Asp222 provides a closer interaction with penicillin G than Ala237 in TEM-1.

There are only three macrostates generated for the active site of DD-transpeptidase with penicillin G (Figure 12D). The most probable transition pathway is going through the sole intermediate state. The similarity among the three macrostates of DD-transpeptidase active site (Figure 13) agrees with the observation of the low flexibility of DD-transpeptidase in the reactant state. This is similar to the active site of TOHO-1. Upon the ligand binding, an active site residue Tyr159 has a rotational motion from the initial state to the intermediate state. In the end state, Tyr159 returns to the conformation as in the initial state.

Both the active sites of TEM-1 and PBP-A show a significant flexibility with multiple transition pathways connecting the bound and unbound states. The TEM-1 and PBP-A have similar t-ICA distributions, and TOHO-1 and DD-transpeptidase simulations have similar narrow arched t-ICA surfaces, but the overall distributions of TEM-1 and TOHO-1 are similar to each other, and PBP-A shares similar layouts with DD-transpeptidase.

### 3.3. Atomic Distance

**Figure 14:**
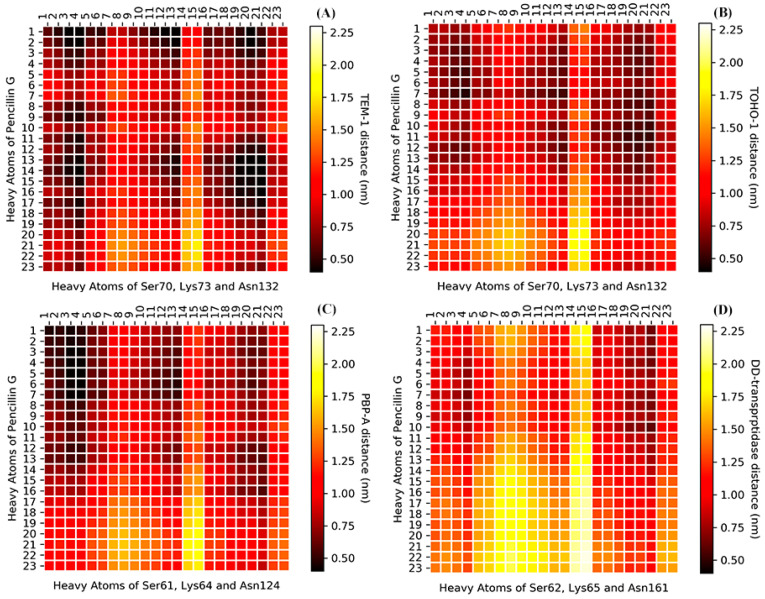
The heat-map of averaged distance (nm) among the heavy atoms of penicillin G and three conserved residues at the active site (**A**) TEM-1: Ser70, Lys73 and Asn132; (**B**) TOHO-1: Ser70, Lys73 and Asn132; (**C**) PBP-A: Ser61, Lys64 and Asn124; (**D**) DD-transpeptidase: Ser62, Lys65 and Asn161. The color bars are listed on the right of the heat map from dark red to light yellow. (The atom numbers of both heavy atoms of penicillin G and three residues are listed in Supplementary Scheme S1, Tables S1 and S2).

**Figure 15:**
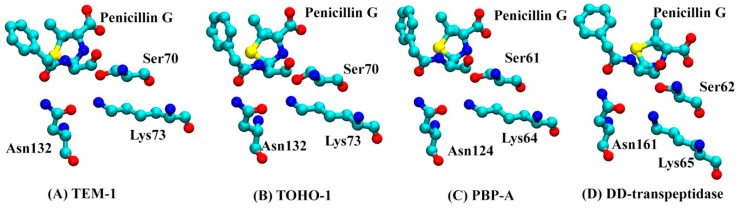
The structures of penicillin G and three conserved residues at active site (Serine, Lysine and Asparagine) in (**A**) TEM-1: Ser70, Lys73 and Asn132; (**B**) TOHO-1: Ser70, Lys73 and Asn132; (**C**) PBP-A: Ser61, Lys64 and Asn124; (**D**) DD-transpeptidase: Ser62, Lys65 and Asn161. The hydrogen atoms are not plotted for clarity.

The analyses of whole protein simulations show that the TEM-1 and TOHO-1 as Class A β-lactamase share similar overall dynamical behaviors, and PBP-A and DD-transpeptidase as PBPs share similar overall dynamical behaviors. But the above analyses of active site dynamics reveal that TEM-1 and PBP-A active sites are more flexible than the active sites of TOHO-1 and DD-transpeptidase. As pointed out earlier in this study, TEM-1, TOHO-1, PBP-A and DD-transpeptidase share three unique residues, including serine, lysine and asparagine, at their active site. [4,5,13,15] To further compare the dynamical behaviors related to their catalysis, the averaged distances among heavy atoms of three residues (in total 23 heavy atoms) and penicillin G (23 heavy atoms) are illustrated as a heat map in Figure 14. The structures of penicillin G and three conserved residues in active site are shown in Figure 15. All the atom numbers on penicillin G, serine, lysine and asparagine structures are listed in Supplementary Scheme S1. 

The darker color means a short distance, and lighter color represents a long distance. The distance heat map could be viewed as a 2D fingerprint for dynamics of this three residues triad in each protein. The bird eye views of TEM-1, TOHO-1 and PBP-A are similar. Although all four heatmaps display overall similar pattern, the heatmaps of TEM-1 and PBP-A are more similar to each other, while the heatmaps of TOHO-1 and DD-transpeptidase are more similar to each other.

### 3.4. Configurational Entropy

**Figure 16:**
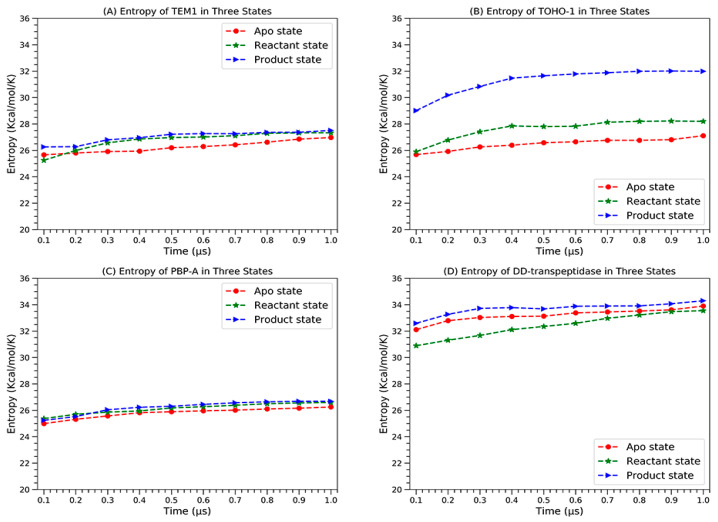
The configurational entropies of four proteins in apo state (red circles), reactant state (green stars) and product state (blue triangles), (**A**) TEM-1, (**B**) TOHO-1, (**C**) PBP-A and (**D**) DD-transpeptidase.

The configurational entropies of four proteins based on the simulations in different states are calculated and plotted in Figure 16. 

All the proteins display clear convergence tendency in each state. TEM-1 and PBP-A are similar to each other in a way that the apo state has the lowest entropy and the reactant and product states have very close entropies. TOHO-1 and DD-transpeptidase are significantly different. The apo state of TOHO-1 also has the lowest entropy. The entropy of TOHO-1 reactant is marginally higher than the apo state. But the entropy of TOHO-1 product state is significantly higher than both apo and reactant state. Therefore, the entropy could be a main driving factor for the TOHO-1 catalysis against various ligands. Different from other three proteins, DD-transpeptidase reactant state has the lowest entropy. Both reactant and product states entropies are only slightly higher with the product state entropy as the highest. Overall, it is unlikely that entropy plays dominant role in the functions of TEM-1, PBP-A and DD-transpeptidase.

## 4. Discussion

In this study, TEM-1, TOHO-1, PBP-A and DD-transpeptidase in the apo, reactant, product states were subjected to MD simulations and detailed analyses. Their dynamical behaviors are impacted differently by the presence of penicillin G as a ligand in the reactant state and the hydrolyzed penicillin G as a ligand in the product state. Although the catalytic and dynamic mechanisms of *β*-lactamases and PBPs in reactant state were investigated intensively, the importance of dynamic behaviors of *β*-lactamases and PBPs in product state is still underestimated. It has been proposed that DD-transpeptidase could be closely related to a common ancestor of modern PBPs and *β*-lactamases [23]. The current study provides dynamical information related to the evolutionary relations between *β*-lactamases and PBPs represented by the target four proteins.

A common Ω loop at the active site for class A *β*-lactamases has been recognized as a crucial structure for their catalysis against antibiotics [5,62]. One study of TEM-1 using MD simulations and NMR showed that TEM-1 is a rigid structure. The main function of Ω loop is to broaden the catalytic cavity through its fluctuation [63]. Our study provides additional insight into the dynamical behavior of TEM-1 Ω loop as it displays more flexibility in the product state than in the apo and reactant states (Figure 5). Meanwhile, the residues 124 to 134 region also shows significant conformational changes only in TEM-1 product state, and is more stable in reactant and apo state. Therefore, this region could be as a potential mutagenesis position to increase the flexibility of Ω loop. This unique feature of Ω loop is also shared with TOHO-1 (Figure 6). However, both PBP-A and DD-transpeptidase do not show this particular feature of Ω loop. For both proteins, the Ω loop or the structure corresponding to the Ω loop do not show high flexibility in the product states (Figure 9 and Figure 11). Therefore, it could be speculated that the high Ω loop flexibility in the product state could be one of key properties of *β*-lactamases developed during the evolution from ancient PBPs. A more general observation of these four proteins is that both the apo and reactant states of TEM-1 and TOHO-1 have similar flexibility which is lower than their product states. But all three states of PBP-A and DD-transpeptidase have similar flexibility. Considering the catalytic function of *β*-lactamases against antibiotics, it could be suggested that higher flexibility in the product state is auxiliary to maintain an appropriate turnover rate.

Residues 218 to 224 form a helix in TEM-1 and is referred to as helix 11. This helix 11 is proposed as an allosteric site in TEM-1 related to its thermal stability [64]. A wider range of residues 216 to 228 enclosing helix 11 display significant flexibility in all three states of TEM-1. In both reactant and product states, this flexible region extends to residue 212 towards the catalytic active site of TEM-1. This observation also supports the allosteric behavior of the helix 11. The Leu220 in this region was proposed to interact with substrates to improve binding. The Val216 also has an auxiliary role to anchor a structurally conserved water molecule [65,66]. Therefore, the region of residues 216 to 228 plays a critical role with substrate binding. In the reactant and product state, this extended flexible region (include up to residue 212) reveals that the dynamics of residues 212 to 215 are impacted by substrate binding interactions. Therefore, residues 212 to 215 could be potential mutagenesis targets to alter the catalytic activities of this protein. Ser70 is a critical residue for TEM-1 catalytic activity, and is located on the loop of residues 64 to 74. The Ser70 is covalently bound to the ring opening intermediate of Penicillin G as a product of the acylation step [4,26]. Interestingly, the loop of residues 64 to 74 display significant flexibility only in the reactant state of TEM-1 (Figure 5), showing its unique role for catalysis in the reactant state.

In TOHO-1, residues 234, 235, and 236 are recognized as the catalytically important KTG sequence [5,67]. Residues 215 to 240 display significant flexibility only in the reactant state of TOHO-1 (Figure 7), also showing its unique role for catalysis in the reactant state. A Ser237Ala mutation in this region can significantly change the catalytic efficiency of this protein. Besides, the residue 240 could affect catalytic efficiencies of many substrates [67]. Furthermore, we propose that other residues in the region of residues 215 to 240 could also serve as mutagenesis targets to adjust the catalytic efficiencies of TOHO-1 against various antibiotics. As an extended-spectrum β-lactamase, TOHO-1 does show higher catalytic activities against more antibiotics than TEM-1. Our analysis shows that TOHO-1 in its reactant state display the higher flexibility than both the apo and product states, which could be correlated with the wide range of ligands profile associated with TOHO-1.

Comparing to other extended-spectrum β-lactamases, TOHO-1 contains a unique residue Arg274 on residues 266 to 288 helix. When binding with penicillin G as a substrate, Arg274 was forced out of the active site since it obstructs the binding pocket of TOHO-1 [5]. This could be one of the reasons that residues 266 to 288 helix display significant flexibility in the reactant state of TOHO-1. Also in TOHO-1, residue Tyr105 was reported to be involved in active site binding with substrate [68]. It was also reported in the same study that Tyr105 displays a single conformation in apo state and two conformations in complexed state. Tyr105 is located on the loop region of residues 97 to 110, which displays significant flexibility only in the product state (Figure 6C).

However, not all the observations about TEM-1 and TOHO-1 in this study agree with the experimental study well. An NMR study of TEM-1 in its apo state identifies residues 124 to 134 with important chemical shift difference [69]. This region, however, displays more prominent flexibility in the product state than in the reactant and apo states (Figure 5). The increased flexibility is reflected in one of the macrostates of TEM-1 in product state (Supplementary Figure S12), and is induced by the binding with the penicillin G hydrolysis product. In addition, the overall RMSFs on total 1 μs simulations of TEM-1 in apo, reactant and product states show similar flexibilities (Supplementary Figure S6). Residues 124 to 134 indeed have fluctuations in TEM-1 apo state, which corresponds to the chemical shift difference in NMR study. However, the flexibility is more observable in the macrostate 2 of product state.

In PBP-A, two loops forming the active site, residues 96 to 108 and Ω loop (residues 154 to 164) display significant flexibilities in all functional states (Figure 9). It was reported that the loop of residues 96 to 108 in PBP-A displays different conformations from the corresponding loop of residues 100 to 115 in TEM-1 [13]. Specifically, Glu104 and Tyr105 in TEM-1 are not well aligned with the equivalent residues Glu96 and Ala97 with an amino acid insertion in PBP-A. This structural difference could be the main reason for the difference of dynamical behavior between PBP-A and TEM-1, which may contribute to the significant differences between the catalytic activity and profiles of these two enzymes.

DD-transpeptidase as the other PBP in this study displays a similar dynamical behavior to PBP-A with similar flexibilities of key secondary structures around the active site. Both Ser62 and Lys65 in DD-transpeptidase are catalytically important residues [15]. The loop of residues 41 to 63 containing residue Ser62 shows moderate flexibility only in the reactant state. In addition, Tyr159 was also proposed as a key residue in acylation step [70,71]. The loop of residues 147 to 163 shows a moderate flexibility only in the product state.

TEM-1 and TOHO-1 in reactant state have increased flexibilities of the residues in the active site than in the apo state, because of the interactions between penicillin G and residues in active site. Starting with the binding with the cavity of protein, the penicillin G ligand gradually leaves the active site after long time simulations, resulting in the observed conformations in initial states, intermediate states, end states and trapped states (Figure 13). Unlike β-lactamases, both PBP-A and DD-transpeptidase do not catalyze wide range of hydrolysis reactions against various antibiotics. Therefore, these enzymes do not display significantly different flexibility among apo, reactant, and product states. In addition, due to the larger size of DD-transpeptidase than other three proteins, the binding with a ligand may lead to smaller impact on protein flexibility.

Further analysis of active site structures combining with the penicillin G as a ligand leads to an unexpected similarity between TEM-1 and PBP-A as well as between TOHO-1 and DD-transpeptidase. We propose that the dynamic properties of the catalytic cavity of TEM-1 are closer to PBP-A than TOHO-1 in terms of their evolution. And the catalysis related dynamics of TOHO-1 has a closer relationship with DD-transpeptidase than with TEM-1. Additional analysis of three residues serine, lysine and asparagine at active site shared by all four proteins leads to some similarities among TEM-1, TOHO-1, and PBP-A, other than DD-transpeptidase. Given that the crystal structures of TEM-1, TOHO-1, and PBP-A are aligned well to each other, while DD-transpeptidase has numerous sequence insertions comparing to the other three proteins (Figure 2), these comparisons do shed light onto the relations among dynamical behaviors of these proteins in different functional states and the functional and evolutionary relations among class A β-lactamases and PBPs.

## 5. Conclusions

In this study, the hidden Markov state model was used to analyze the molecular dynamics simulations of two class A β-lactamases, TEM-1 and TOHO-1, and two penicillin binding proteins, PBP-A and DD-transpeptidase. Both principal component analysis and time-lagged independent component analysis were employed as dimensionality reduction methods for the projection of simulations onto reduced dimensions of space. The analysis of dynamical behaviors of overall structures of four proteins agrees with the overall structural comparison in that the key loop structures around the active site in TEM-1 and TOHO-1 displays lower flexibility in the apo and reactant states than in the product state. Likely due to its wider spectrum of ligands, TOHO-1 displays higher overall flexibility in its reactant state than in the apo and product states. Both PBP-A and DD-transpeptidase display consistent flexibility in all three states, agreeing with their specific catalytic functions. Additional analysis of dynamical behavior of active sites complexed with penicillin G in these four proteins reveals that the active sites are rather flexible in TEM-1 and PBP-A with multiple transition pathways between bound and unbound states. Further analysis of three key catalytic residues shared among four proteins indicates similarity of dynamical property among TEM-1, TOHO-1, and PBP-A. All the dynamics analyses presented in this study show the complication of evolutionary relations between β-lactamases and PBPs, and support the notion that protein dynamics may play a significant role in and are characterized by the catalytic functions of these enzymes.

## Figures and Tables

**Table 1:** The residues in active site of TEM-1, TOHO-1, PBP-A and DD-transpeptidase.

TEM-1	TOHO-1	PBP-A	DD-Transpeptidase
S70	S70	S61	S62
K73	K73	K64	K65
S130	S130	S122	Y159
N132	N132	N124	N161
N166	A166	L158	A237
K234	K234	K219	H298
S235	T235	T220	T299
A237	S237	D222	T301
G244	N245	G228	T307

## Supplementary Materials

The following are available online at https://www.mdpi.com/1099-4300/21/11/1130/s1, Figure S1: Estimated relaxation timescale based on different lag time for TEM-1. (A) Apostate simulations; (B) Reactant state; (C) Product state. The relaxation timescales are estimated based on transition probabilities among different microstates regarding with the different lag time as interval for analysis, Figure S2: Estimated relaxation timescale based on different lag time for TOHO-1. (A) Apo state simulations; (B) Reactant state; (C) Product state. The relaxation timescales are estimated based on transition probabilities among different microstates regarding with the different lag time as interval for analysis, Figure S3: Estimated relaxation timescale based on different lag time for PBP-A. (A) Apo state simulations; (B) Reactant state; (C) Product state. The relaxation timescales are estimated based on transition probabilities among different microstates regarding with the different lag time as interval for analysis, Figure S4: Estimated relaxation timescale based on different lag time for DD-transpeptidase. (A) Apo state simulations; (B) Reactant state; (C) Product state. The relaxation timescales are estimated based on transition probabilities among different microstates regarding with the different lag time as interval for analysis, Figure S5: Estimated relaxation timescales based on different lag time for active site binding with penicillin G in reactant states of: (A) TEM-1; (B) TOHO-1; (C) PBP-A; (D) DDtranspeptidase. The relaxation timescales are estimated based on transition probabilities among different microstates regarding with the different lag time as interval for analysis, Figure S6: The RMSFs for 1 μs TEM-1 simulations in apo, reactant and product states, Figure S7: The RMSFs for 1 μs TOHO-1 simulations in apo, reactant and product states, Figure S8: The RMSFs for 1 μs PBP-A simulations in apo, reactant and product states, Figure S9: The RMSFs for 1 μs DD-transpeptidase simulations in apo, reactant and product states, Figure S10: The RMSFs for three macrostates in TEM-1 apo state simulations. Region A1 represents residues 163 to 178 (Ω loop), Region A2 represents residues 216 to 228, Figure S11: The RMSFs for three macrostates in TEM-1 reactant state simulation. Region A1 represents residues 64 to 74, region A2 represents residues 163 to 178 (Ω loop), region A3 represents residues 212 to 228, and region A4 represents residues 234 to 236, Figure S12: The RMSFs for four macrostates in TEM-1 product state simulations. Region A1 represents residues 124 to 134, region A2 represents residues 163 to 178 (Ω loop), and region A3 represents residues 212 to 228, Figure S13: The RMSFs for three macrostates of TOHO-1 apo state simulations. Region A1 represents residues 27 to 45, and region A2 represents residues 160 to 178 (Ω loop), Figure S14: The RMSFs for four macrostates in TOHO-1 reactant state simulations. Region A1 represents residues 27 to 45, region A2 represents residues 160 to 178 (Ω loop), region A3 represents residues 215 to 240, and region A4 represents residues 266 to 288, Figure S15: The RMSFs for five macrostates in TOHO-1 product state simulations. Region A1 represents residues 97 to 110, and region A2 represents residues 160 to 178 (Ω loop), Figure S16: The RMSFs for four macrostates in PBP-A apo state simulation. Region A1 represents residues 96 to 108, and region A2 represents residues 154 to 164, Figure S17: The RMSFs for four macrostates in PBP-A reactant state simulations. Region A1 represents residues 48 to 64, region A2 represents residues 96 to 106, and region A3 represents residues 154 to 164 (Ω loop), Figure S18: The RMSFs for four macrostates in PBP-A product state simulations. Region A1 represents residues 96 to 108, and region A2 represents residues 154 to 164 (Ω loop), Figure S19: The RMSFs for four macrstates in DD-transpeptidase apo state simulations. Region A1 represents residues 117 to 141, region A2 represents residues 227 to 243, and region A3 represents residues 273 to 279, Figure S20: The RMSFs for four macrostates in DD-transpeptidase reactant state simulations. Region A1 represents residues 41 to 63, region A2 represents residues 117 to 141, and region A3 represents residues 170 to 183, Figure S21: The RMSFs for four macrostates in DD-transpeptidase product state simulations. Region A1 represents residues 117 to 141, region A2 represents residues 147 to 163, region A3 represents residues 225 to 239, and region A4 represents residues 271 to 279, Scheme S1: Penicillin G and three residues at active site (Serine, Lysine and Asparagine) shared by TEM-1, TOHO-1, PBP-A and DD-transpeptidase. The atomic symbol with sequence numbers are corresponding to the symbol used atomic distances in heat map, Table S1: The atomic numbers used in averaged distance heatmap for the heavy atoms of Serine, Lysine and Asparagine (1-23) shared among all four proteins, Table S2: The atomic numbers used in averaged distance heatmap, the heavy atoms in penicillin G (1-23).
