# Supplementary material for: Dynamical Behavior of β-Lactamases and Penicillin- Binding Proteins in Different Functional States and Its Potential Role in Evolution
Source: Entropy (Basel). 2019 Nov 19;21(11):1130. doi: 10.3390/e21111130 (PMC7514474; doi:10.3390/e21111130)
Supplement: Supplementary file 1 [file entropy-21-01130-s001.pdf]

Supplementary Material

Dynamical Behavior of β-Lactamases and Penicillin-Binding Proteins in Different Functional States and Its Potential Role in Evolution

Feng Wang,[1] Hongyu Zhou,[1] Xinlei Wang,[2] and Peng Tao [1,*]

[1] Department of Chemistry, Center for Drug Discovery, Design, and Delivery (CD4), Center for Scientific Computation, Southern Methodist University, Dallas, Texas 75275, United States
[2] Department of Statistical Science, Southern Methodist University, Dallas, Texas 75275, United States
* Correspondence: ptao@smu.edu

1 Preprocessing Trajectories for Principal Components Analysis

The trajectories of TOHO-1, PBP-A, DD-transpeptidase in three states are superimposed with TEM-1 structures (apo, reactant and product states) as reference. For PBP-A and DD-transpeptidase some deletions and insertions exist in the structural alignment. So α carbon of the residues in PBP-A and DD-transpeptidase aligned with TEM-1 were selected and used on superimposing process. For TOHO-1, all α carbons were selected.

PBP-A structure in apo, reactant and product states are all superimposed with the TEM-1 structure as the reference. The residues 26-27, 28-36, 41-52, 57-113, 114-168, 177-215, 220-240, 242-249, 251-254 and 271-288 in TEM-1 structure are used as the reference. The corresponding residues in PBP-A are residues 19-20, 23-31, 33-44, 48-104, 106-160, 163-201, 205-225, 226-233, 237-240 and 254-271.

DD-transpeptidase structure in apo, reactant and product states are all superimposed with the TEM-1 structure as the reference. The residues 26-28, 30-40, 41-42, 43-50, 54-55, 57, 58-60, 61-95, 96, 97, 113-114, 115, 117-125, 128, 129-142, 143, 144-155, 156-163, 164, 165-168, 179-209, 210, 211, 212, 217, 222, 225-240, 242-250, 254-269, 270-281, 285-288 on TEM-1 structure are used as the reference. The corresponding residues on DD-transpeptidase are residues 5-7, 8-18, 22-23, 25-32, 33-34, 35, 37-39, 53-87, 93,95, 96-97, 99, 100-108, 109, 158-171, 177, 179-190, 192-199, 232, 236-239, 240-270, 282, 284, 286, 287, 288, 289-304, 305-313 and 314-329.

2. The implied timescales using different lag times for TEM-1, TOHO-1, PBP-A and DD-transpeptidase in apo, reactant and product states

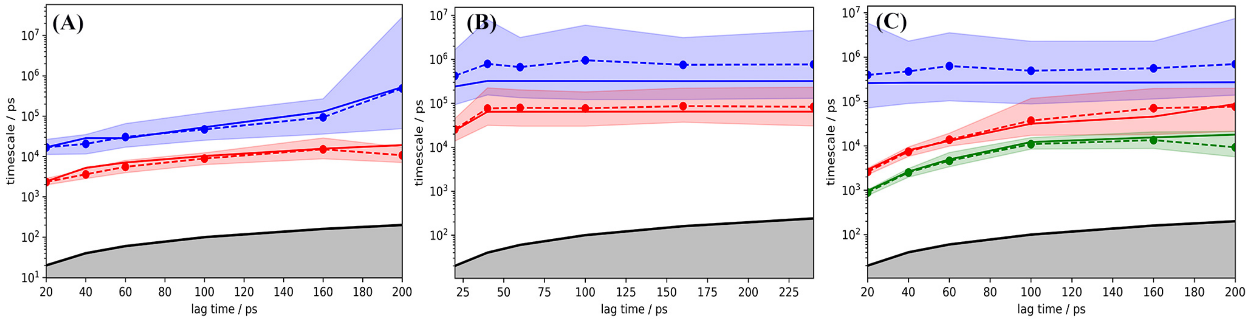

Supplementary Figure 1. Estimated relaxation timescale based on different lag time for TEM-1. (A) Apo state simulations; (B) Reactant state; (C) Product state. The relaxation timescales are estimated based on transition probabilities among different microstates regarding with the different lag time as interval for analysis.

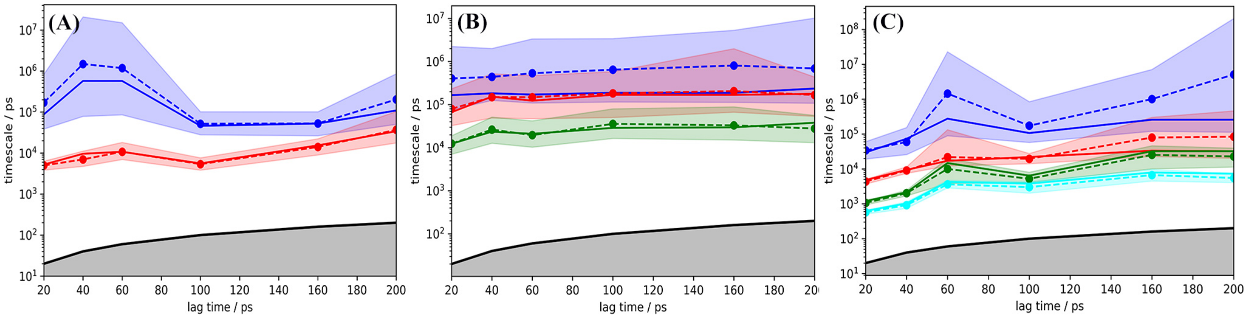

Supplementary Figure 2. Estimated relaxation timescale based on different lag time for TOHO-1. (A) Apo state simulations; (B) Reactant state; (C) Product state. The relaxation timescales are estimated based on transition probabilities among different microstates regarding with the different lag time as interval for analysis.

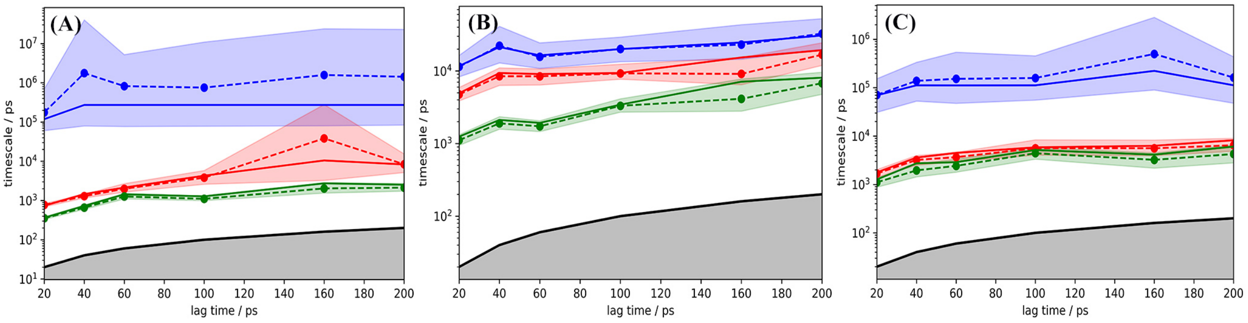

Supplementary Figure 3. Estimated relaxation timescale based on different lag time for PBP-A. (A) Apo state simulations; (B) Reactant state; (C) Product state. The relaxation timescales are estimated based on transition probabilities among different microstates regarding with the different lag time as interval for analysis.

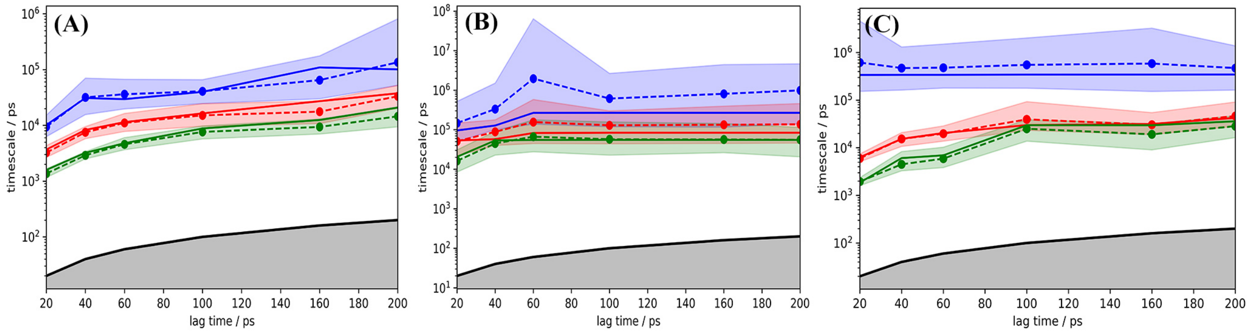

Supplementary Figure 4. Estimated relaxation timescale based on different lag time for DD-transpeptidase. (A) Apo state simulations; (B) Reactant state; (C) Product state. The relaxation timescales are estimated based on transition probabilities among different microstates regarding with the different lag time as interval for analysis.

3. The implied timescales using different lag times for the trajectories of active sites with penicillin G in apo, reactant and product states of TEM-1, TOHO-1, PBP-A and DD-transpeptidase

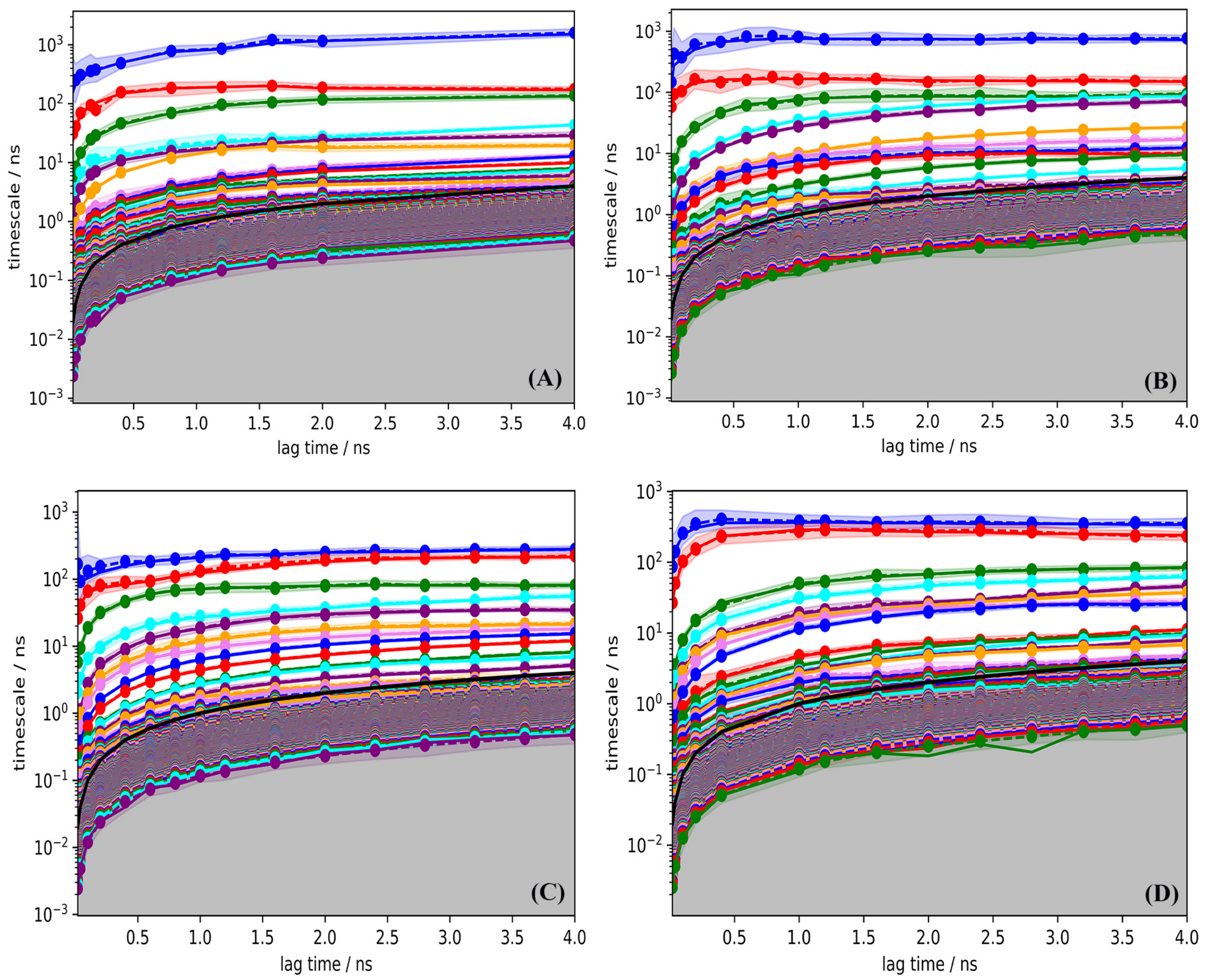

Supplementary Figure 5. Estimated relaxation timescales based on different lag time for active site binding with penicillin G in reactant states of: (A) TEM-1; (B) TOHO-1; (C) PBP-A; (D) DD-transpeptidase. The relaxation timescales are estimated based on transition probabilities among different microstates regarding with the different lag time as interval for analysis.

4. The RMSF of TEM-1, TOHO-1, PBP-A and DD-transpeptidase for 1μs simulations in apo, reactant and product states

The RMSFs are calculated based on 1μs simulation of TEM-1 in apo, reactant and product states. The ensemble of RMSFs for all three functional states are uniform. While several segments on overall structure are different in three states. For example, the residues 56 to 80 and residues 150 to 176 have different amplitudes in fluctuation.

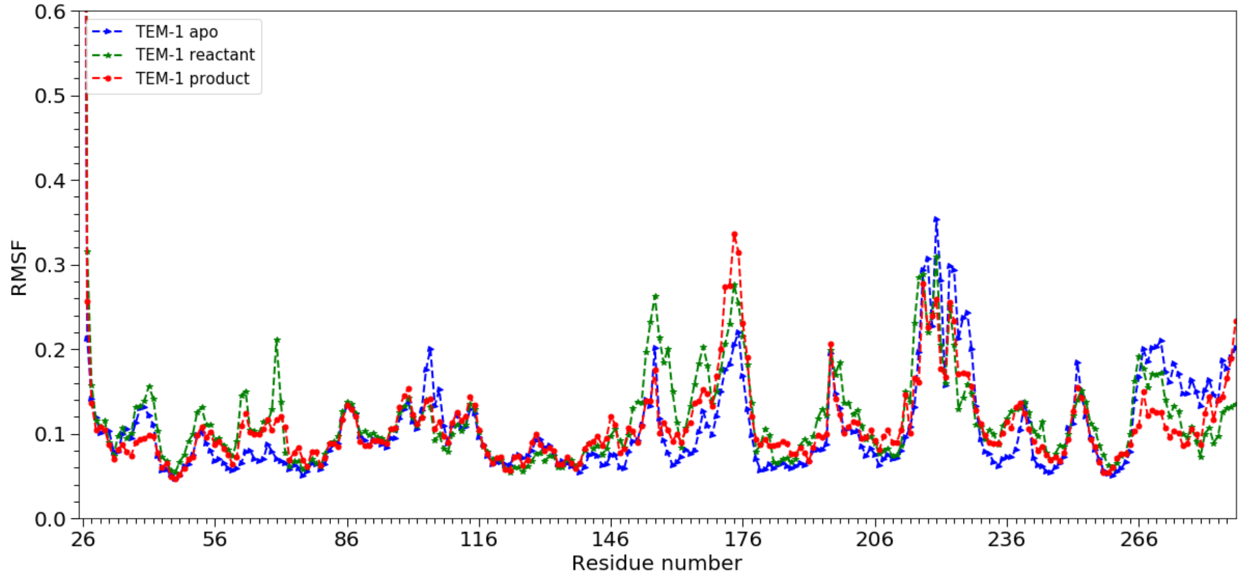

Supplementary Figure 6. The RMSFs for 1μs TEM-1 simulations in apo, reactant and product states.

The RMSF are calculated based on $1\mu s$ simulation of TOHO-1 in apo, reactant and product states. The product state of TOHO-1 has a higher fluctuation than in apo and reactant states and the highest RMSF value is up to 1.6, which indicate that the product state of TOHO-1 is more flexible than in other two states.

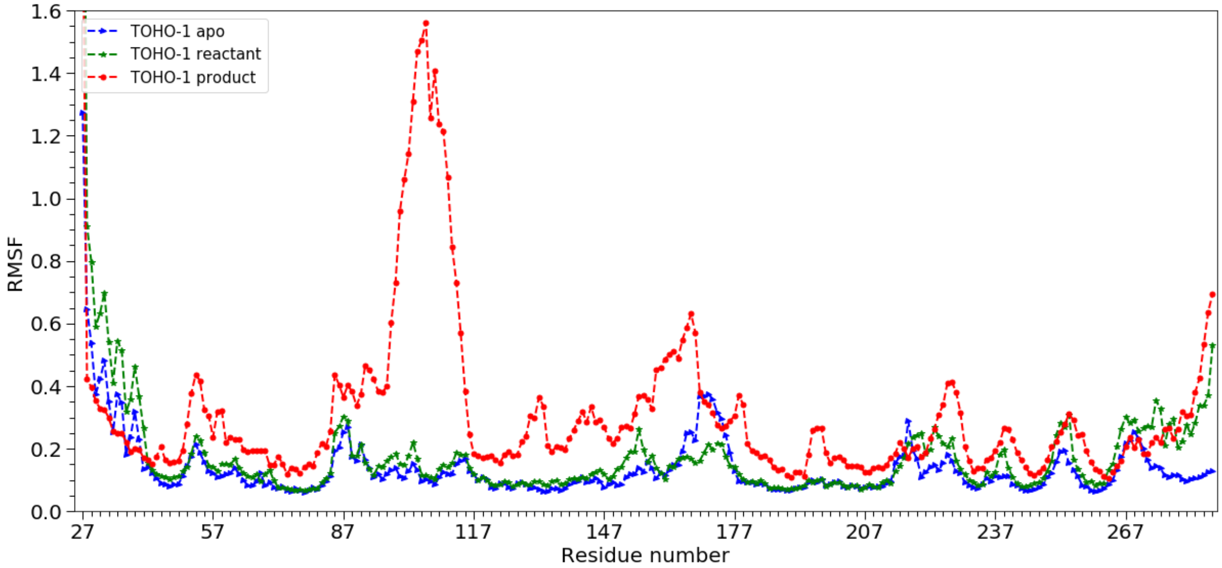

Supplementary Figure 7. The RMSFs for 1μs TOHO-1 simulations in apo, reactant and product states.

The RMSF are calculated based on 1μs simulation of PBP-A in apo, reactant and product states. The ensemble of RMSFs for all three functional states are uniform. Only two segments indicate different fluctuations, including residues 96 to 108 and residues 156 to 164.

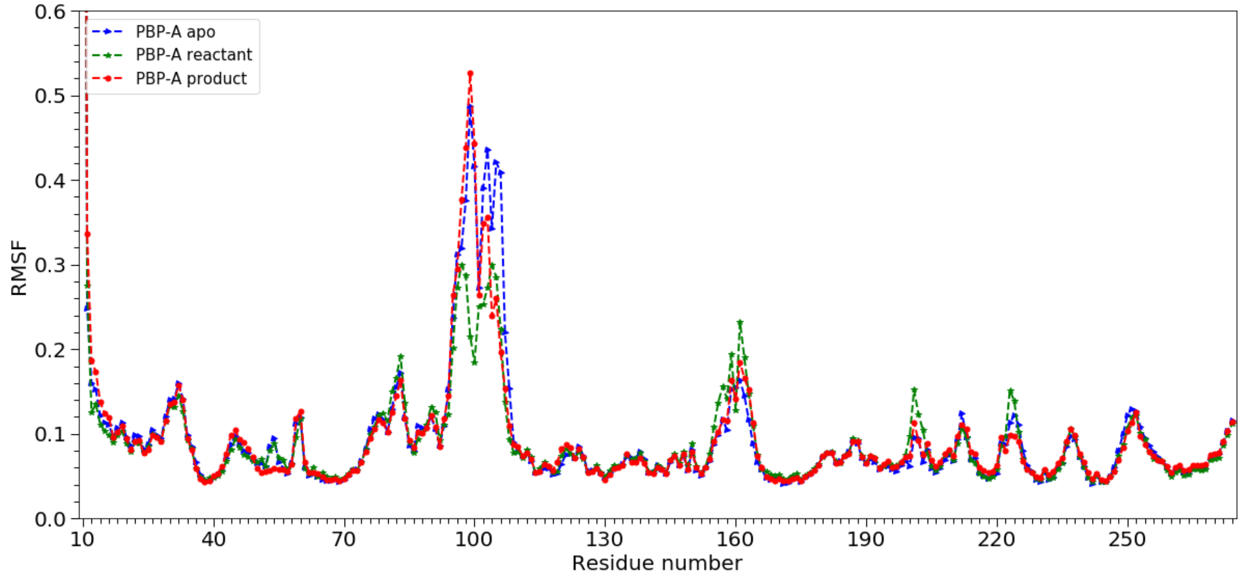

Supplementary Figure 8. The RMSFs for 1μs PBP-A simulations in apo, reactant and product states.

The RMSF are calculated based on $1\mu s$ simulation of DD-transpeptidase in apo, reactant and product states. The reactant state of DD-transpeptidase is more stable than it in apo and product states, which indicates the benzyl penicillin can stabilize the overall structure of DD-transpeptidase.

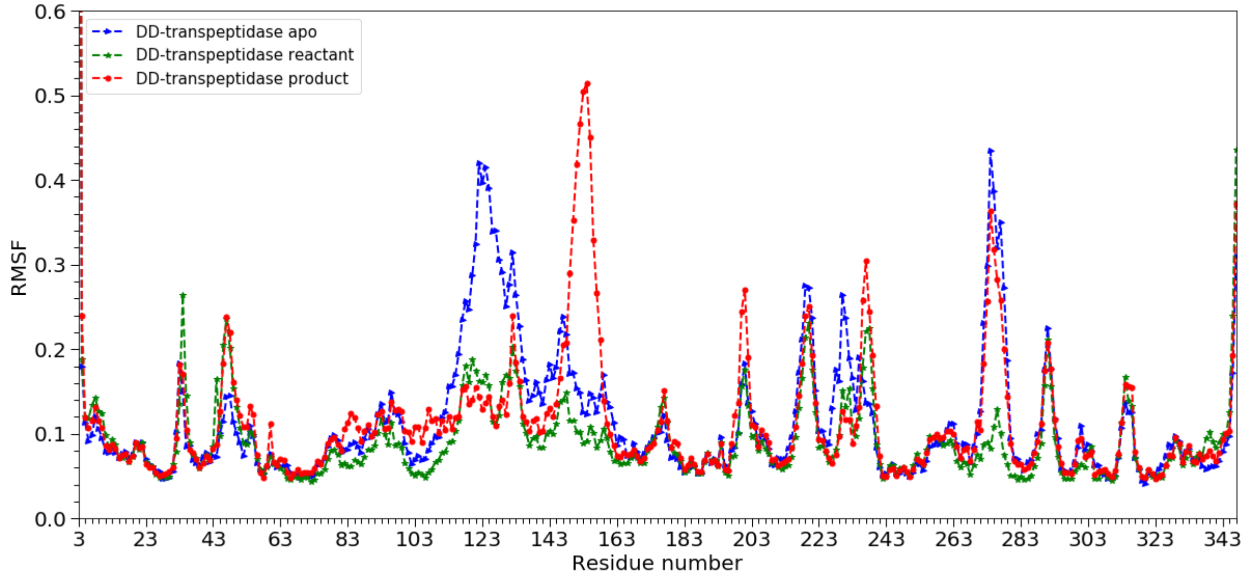

Supplementary Figure 9. The RMSFs for 1μs DD-transpeptidase simulations in apo, reactant and product states.

5. The RMSF of each macrostate for TEM-1, TOHO-1, PBP-A and DD-transpeptidase

In TEM-1 (SI Figure 10-12), TOHO-1 (SI Figure 13-15), PBP-A (SI Figure 16-18) and DD-transpeptidase (SI Figure 19-21), many segments have significantly different fluctuations in different macrostates. Those segments are highlighted in the RMSFs plots.

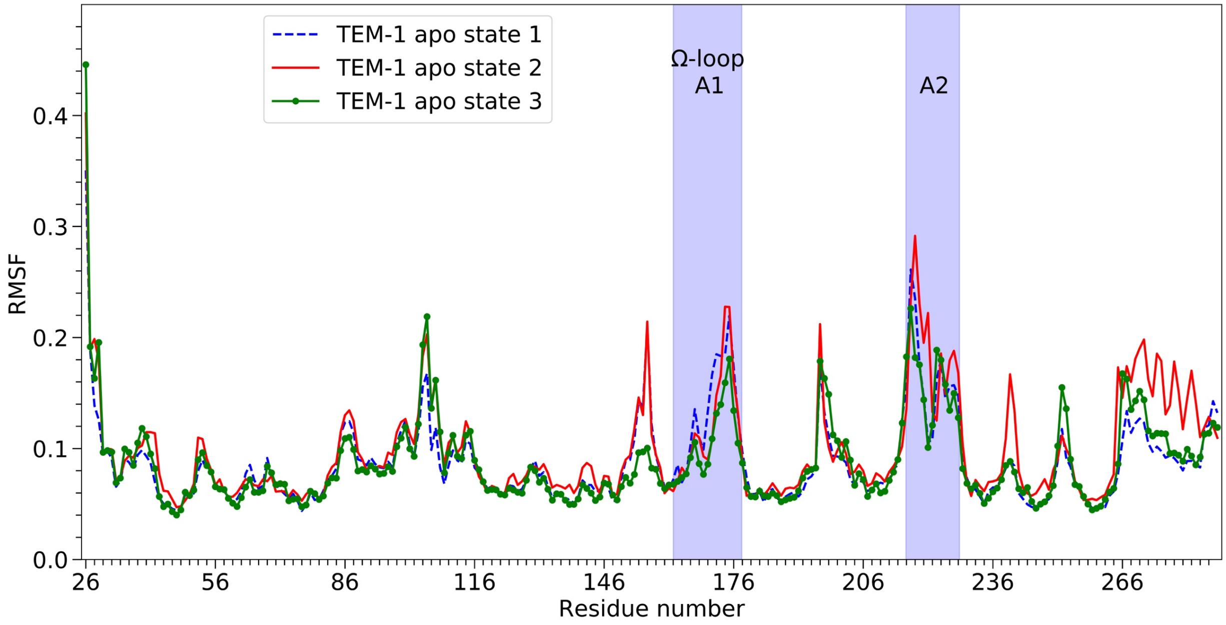

Supplementary Figure 10. The RMSFs for three macrostates in TEM-1 apo state simulations. Region A1 represents residues 163 to 178 (Ω loop), Region A2 represents residues 216 to 228.

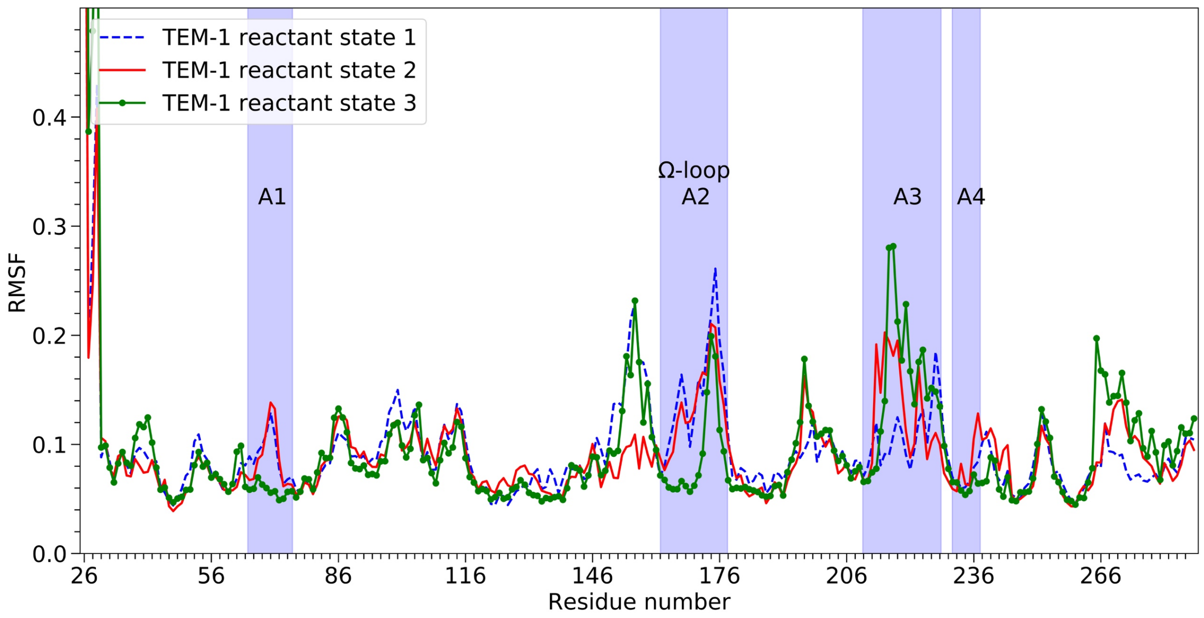

Supplementary Figure 11. The RMSFs for three macrostates in TEM-1 reactant state simulation. Region A1 represents residues 64 to 74, region A2 represents residues 163 to 178 (Ω loop), region A3 represents residues 212 to 228, and region A4 represents residues 234 to 236.

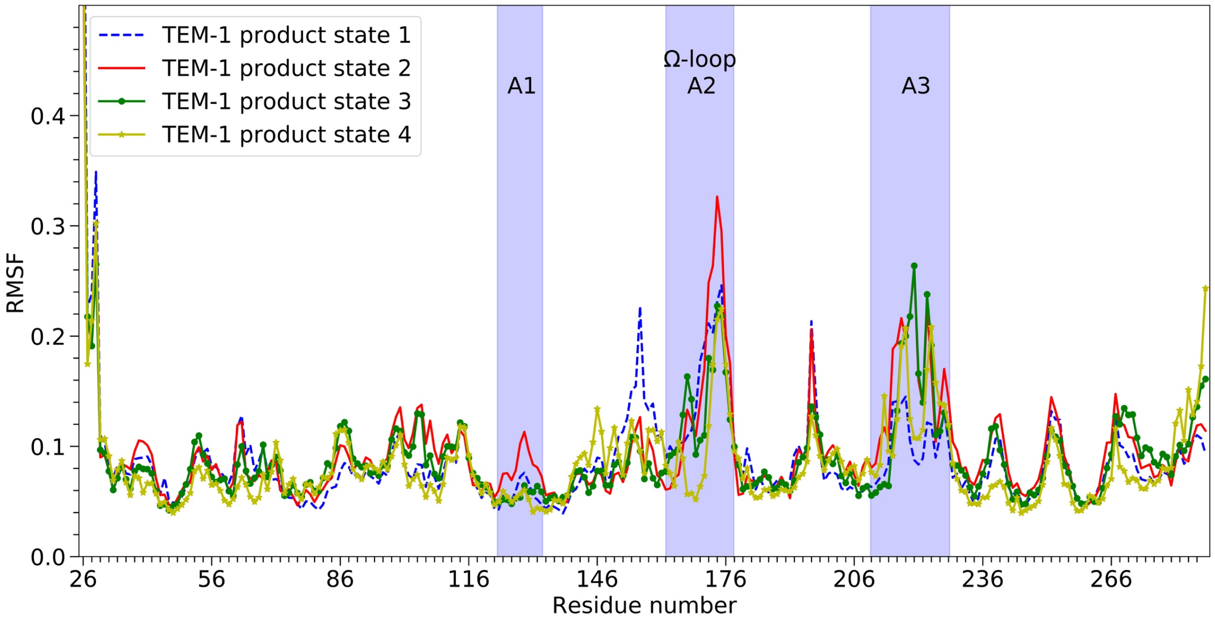

Supplementary Figure 12. The RMSFs for four macrostates in TEM-1 product state simulations. Region A1 represents residues 124 to 134, region A2 represents residues 163 to 178 (Ω loop), and region A3 represents residues 212 to 228

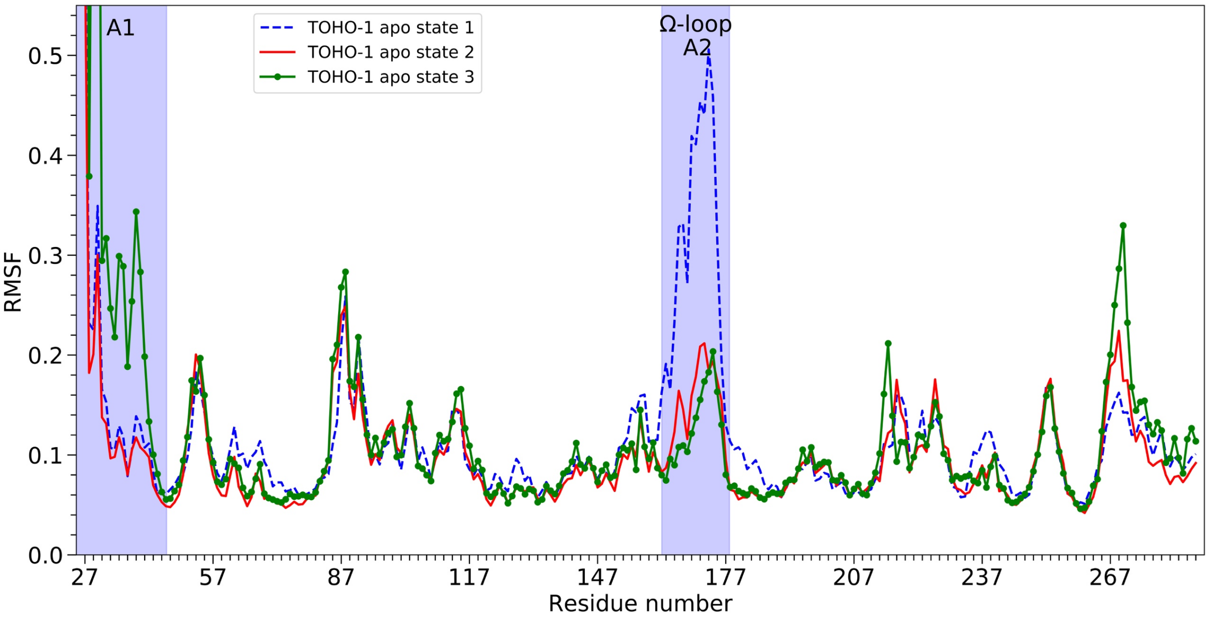

Supplementary Figure 13. The RMSFs for three macrostates of TOHO-1 apo state simulations. Region A1 represents residues 27 to 45, and region A2 represents residues 160 to 178 (Ω loop).

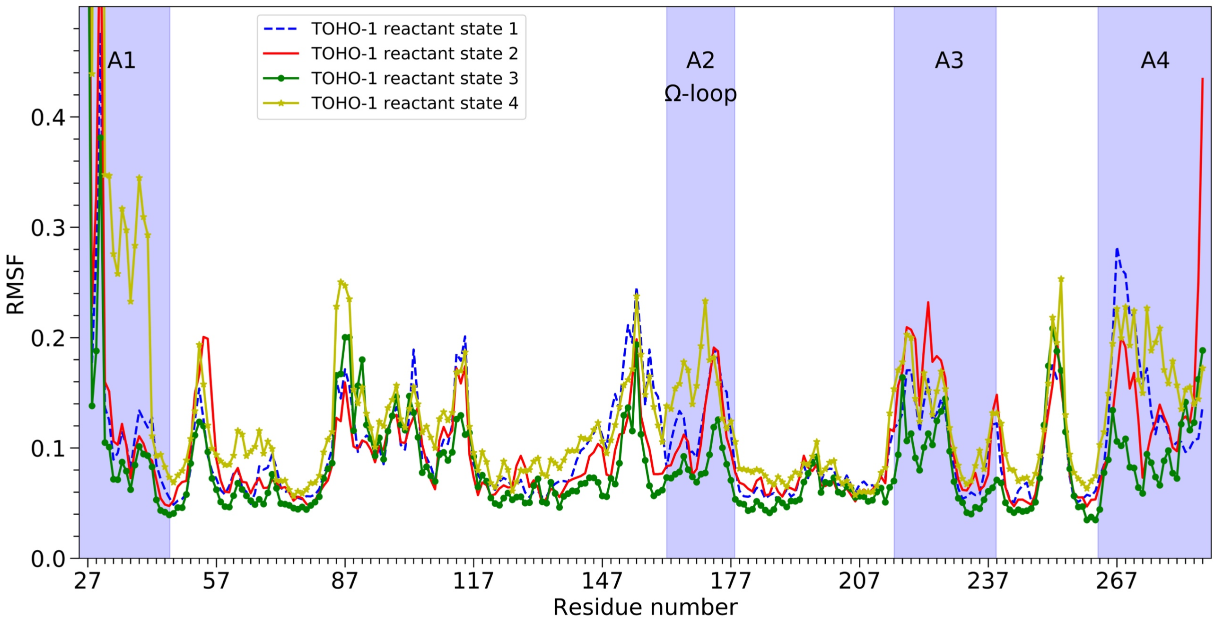

Supplementary Figure 14. The RMSFs for four macrostates in TOHO-1 reactant state simulations. Region A1 represents residues 27 to 45, region A2 represents residues 160 to 178 (Ω loop), region A3 represents residues 215 to 240, and region A4 represents residues 266 to 288.

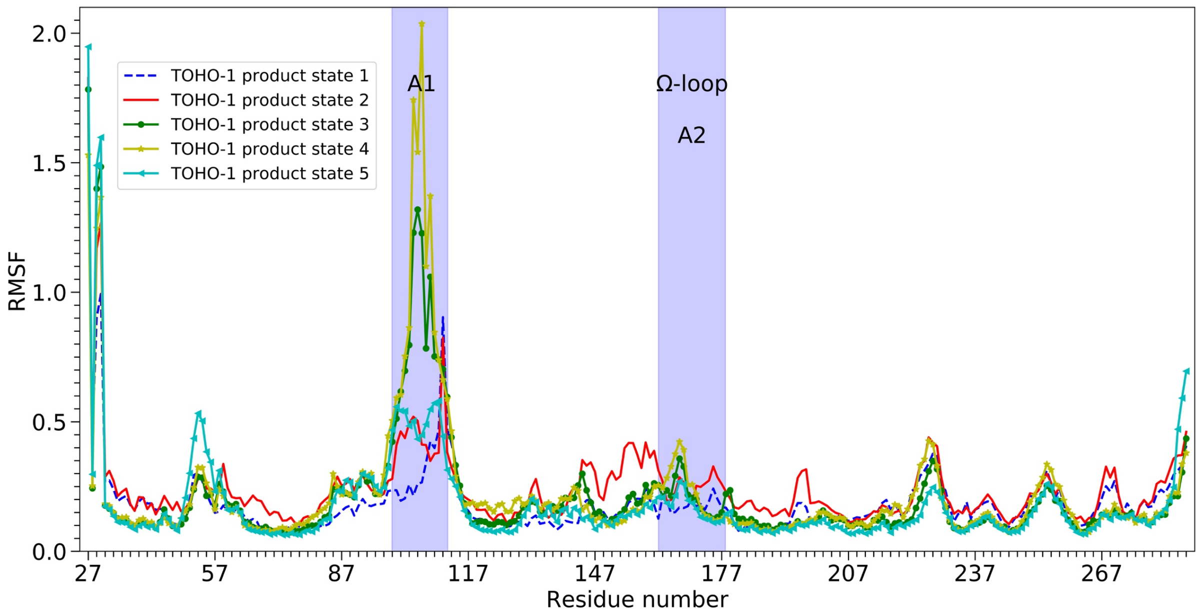

Supplementary Figure 15. The RMSFs for five macrostates in TOHO-1 product state simulations. Region A1 represents residues 97 to 110, and region A2 represents residues 160 to 178 (Ω loop).

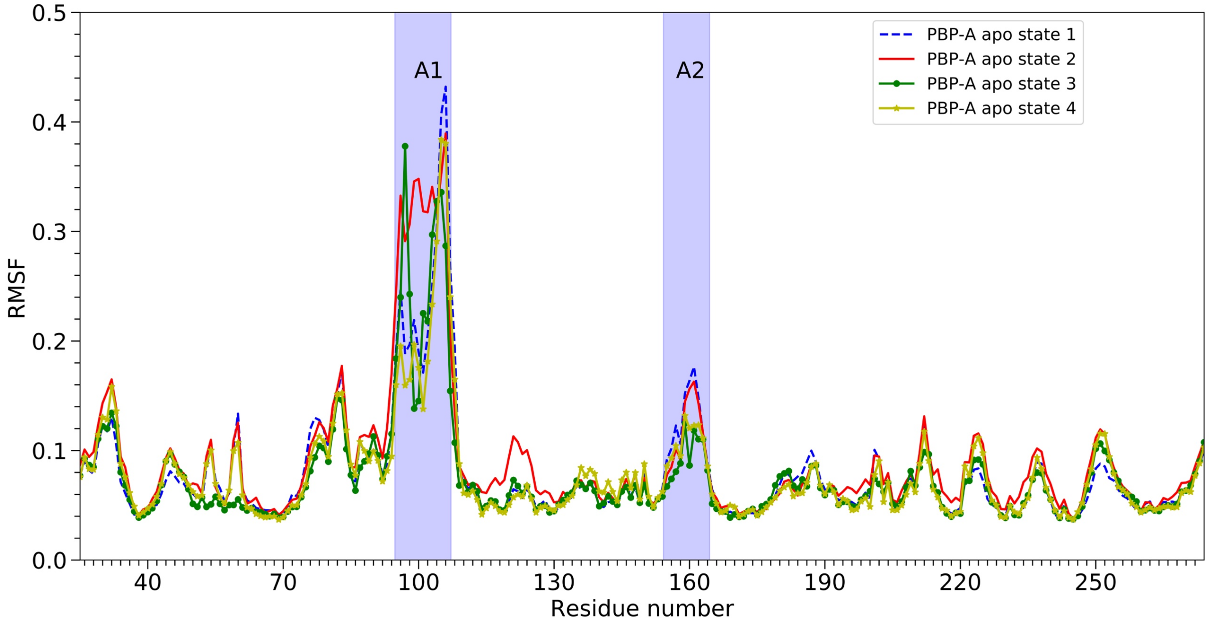

Supplementary Figure 16. The RMSFs for four macrostates in PBP-A apo state simulation. Region A1 represents residues 96 to 108, and region A2 represents residues 154 to 164.

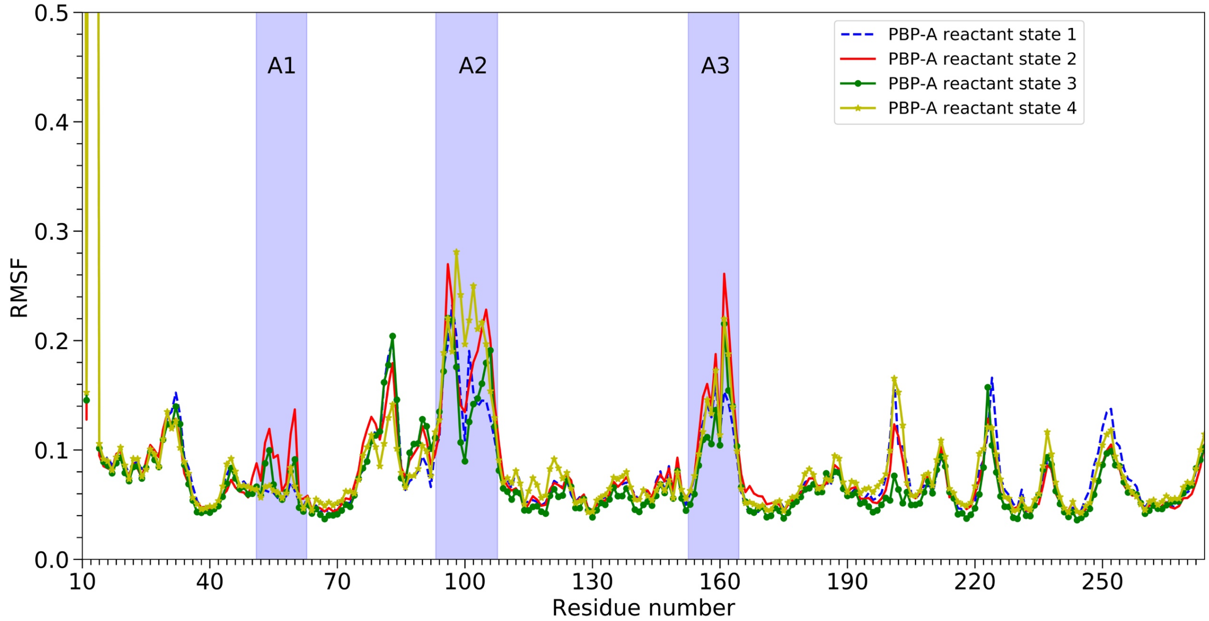

Supplementary Figure 17. The RMSFs for four macrostates in PBP-A reactant state simulations. Region A1 represents residues 48 to 64, region A2 represents residues 96 to 106, and region A3 represents residues 154 to 164 (Ω loop).

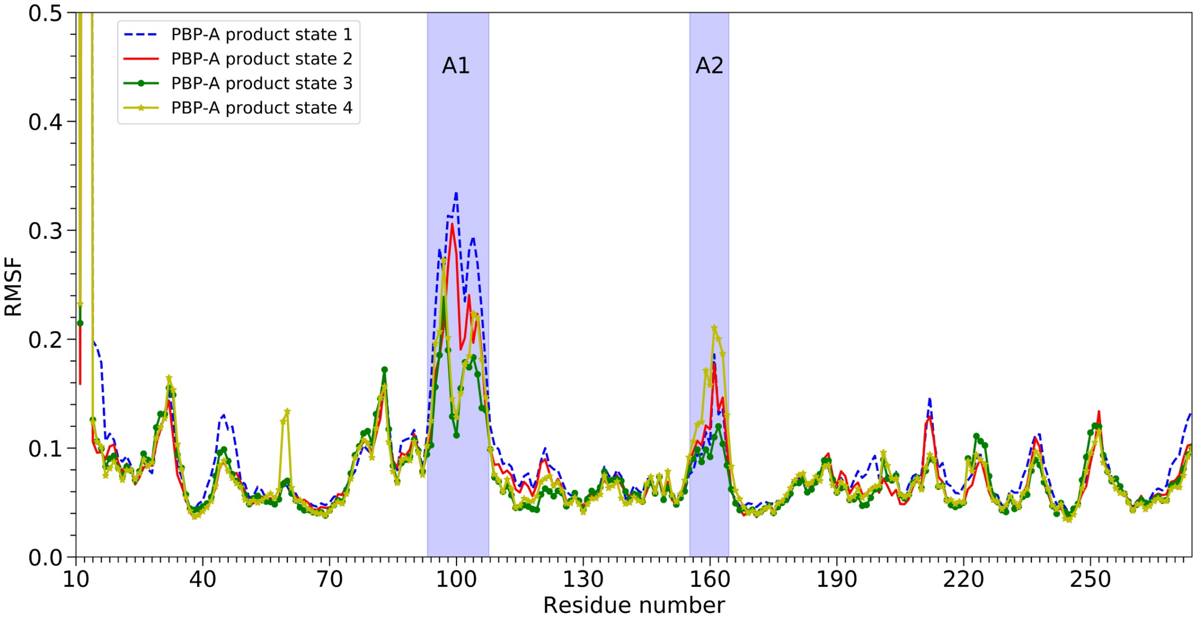

Supplementary Figure 18. The RMSFs for four macrostates in PBP-A product state simulations. Region A1 represents residues 96 to 108, and region A2 represents residues 154 to 164 (Ω loop).

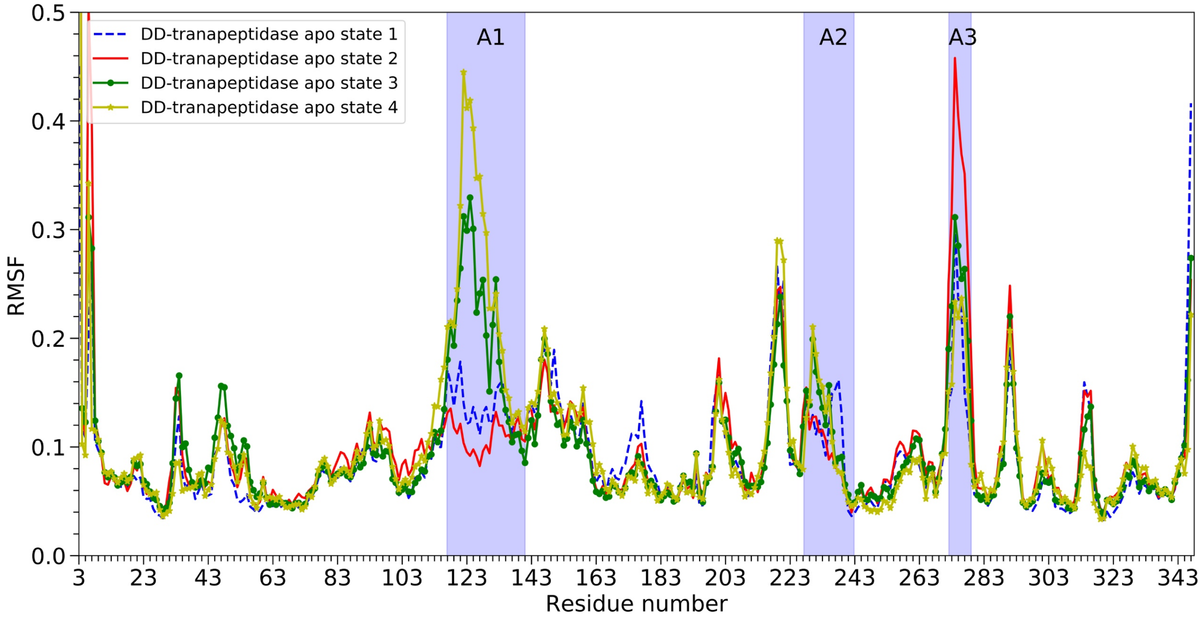

Supplementary Figure 19. The RMSFs for four macrstates in DD-transpeptidase apo state simulations. Region A1 represents residues 117 to 141, region A2 represents residues 227 to 243, and region A3 represents residues 273 to 279.

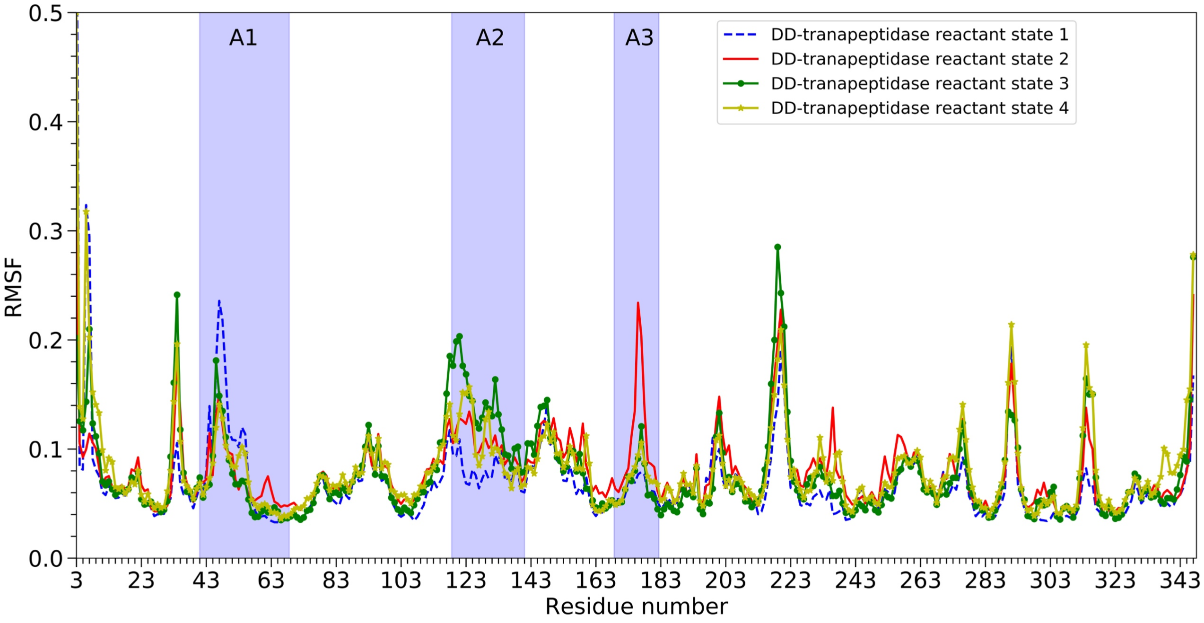

Supplementary Figure 20. The RMSFs for four macrostates in DD-transpeptidase reactant state simulations. Region A1 represents residues 41 to 63, region A2 represents residues 117 to 141, and region A3 represents residues 170 to 183.

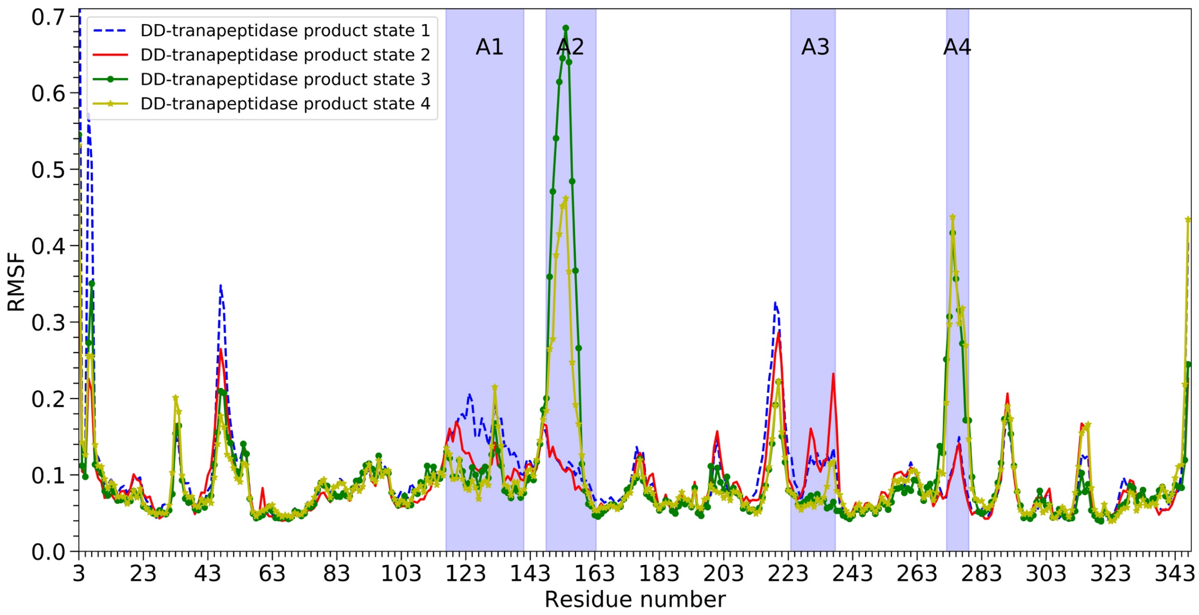

Supplementary Figure 21. The RMSFs for four macrostates in DD-transpeptidase product state simulations. Region A1 represents residues 117 to 141, region A2 represents residues 147 to 163, region A3 represents residues 225 to 239, and region A4 represents residues 271 to 279.

6. The atomic distance analysis

The heavy atoms of penicillin G are ordered as O1, C1, N1, C2, C3, O2, O3, C4, C5, C6, S, C7, C8, N2, C9, O4, C10, C11, C12, C13, C14, C15 and C16 (total 23 atoms from 1-23). The heavy atoms of three residues are ordered as N, C2, C3, O2, C1, O1 in Serine, N1, C2, C3, C4, C5, C6, N2, C1, O in Lysine, N2, C3, C2, C1, O1, N1, C4, O2 in Asparagine (total 23 atoms in three residues from 1-23). The atom numbers labeled on penicillin G and three residues are identical in four proteins.

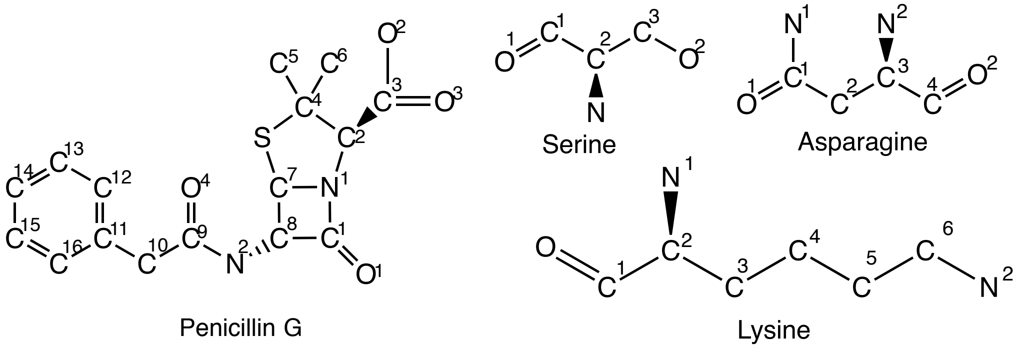

Supplementary Scheme 1. Penicillin G and three residues at active site (Serine, Lysine and Asparagine) shared by TEM-1, TOHO-1, PBP-A and DD-transpeptidase. The atomic symbol with sequence numbers are corresponding to the symbol used atomic distances in heat map.

Supplementary Table 1. The atomic numbers used in averaged distance heatmap for the heavy atoms of Serine, Lysine and Asparagine (1-23) shared among all four proteins.

**Atomic Number**	**Heavy atoms of three residues**	**Residues**
1	N	
2	C2	
3	C3	**Serine**
4	O2	
5	C1	
6	O1	
7	N1	
8	C2	
9	C3	
10	C4	
11	C5	**Lysine**
12	C6	
13	N2	
14	C1	
15	O	
16	N2	
17	C3	
18	C2	
19	C1	
20	O1	**Lysine**
21	N1	
22	C4	
23	O2	

Supplementary Table 2. The atomic numbers used in averaged distance heatmap, the heavy atoms in penicillin G (1-23)

**Atomic Number**	**Heavy atoms of Penicillin G**
1	O1
2	C1
3	N1
4	C2
5	C3
6	O2
7	O3
8	C4
9	C5
10	C6
11	S
12	C7
13	C8
14	N2
15	C9
16	O4
17	C10
18	C11
19	C12
20	C13
21	C14
22	C15
23	C16
